# Global burden of nasopharyngeal carcinoma attributable to alcohol use: a 1990–2021 analysis with projections to 2040

**DOI:** 10.3389/fpubh.2025.1623089

**Published:** 2025-08-07

**Authors:** Zhenyi Lu, Shujun Yang, Mengqi Dai, Guixiang Wu, Fabao Wang, Kai Zhang

**Affiliations:** ^1^Department of Otolaryngology, The First Affiliated Hospital of Anhui Medical University, Hefei, Anhui, China; ^2^Department of Otolaryngology, The Second Affiliated Hospital of Bengbu Medical University, Bengbu, Anhui, China; ^3^The Fifth Clinical School of Anhui Medical University, Fuyang, Anhui, China; ^4^Department of Otolaryngology, The First Affiliated Hospital of Bengbu Medical University, Bengbu, Anhui, China; ^5^Department of Stomatology, The First Affiliated Hospital of Bengbu Medical University, Bengbu, Anhui, China

**Keywords:** nasopharyngeal carcinoma, alcohol-attributable burden, global burden, mortality, disability-adjusted life years

## Abstract

**Background:**

Nasopharyngeal carcinoma attributable to alcohol use (NPC-AU) contributes substantially to global cancer mortality and disability, yet its temporal and geographic patterns remain incompletely described.

**Objectives:**

To assess the global, regional, and national burden of NPC-AU from 1990 to 2021 and project trends through 2040.

**Material and methods:**

Using GBD 2021 data, global, regional, and national age-standardized mortality rates (ASMR) and disability-adjusted life-years rates (ASDR) attributable to alcohol were evaluated. Trends were quantified by average annual percentage change (AAPC) and projections were generated using Bayesian age–period–cohort models.

**Results:**

From 1990 to 2021, global ASMR declined from 0.31 to 0.19 per 100,000 population (AAPC −1.66; 95% CI −1.79 to −1.52) and ASDR fell with an AAPC of −1.72 (95% CI −1.87 to −1.57). Male ASMR decreased from 0.42 to 0.27 per 100,000 (AAPC −1.60), and female ASMR from 0.21 to 0.12 per 100,000 (AAPC −2.25). High-middle SDI regions saw ASMR drop from 0.50 to 0.28 per 100,000 (AAPC −1.97), whereas low-middle SDI regions experienced an increase from 0.09 to 0.11 per 100,000 (AAPC 0.72). Regionally, East Asia's ASMR declined at an AAPC of −2.70, Southern Latin America at −3.13, and Southeast Asia increased at 1.76. Age-specific peaks in ASMR shifted from 55–59 and 65–69 years in 1990 to 65–69 and 70–74 years in 2021. Projections forecast male ASMR of 0.35 per 100,000 (95% UI 0.03–0.67) and female ASMR of 0.02 per 100,000 (95% UI 0.00–0.04) by 2040.

**Conclusions and significance:**

Although global ASMR and ASDR for NPC-AU declined markedly from 1990 to 2021, rising burdens in lower-SDI regions, persistent male predominance, and shifting peaks to older age groups highlight the need for targeted alcohol-control policies and age-tailored screening.

## Introduction

Nasopharyngeal carcinoma (NPC) is recognized as a relatively uncommon but aggressive epithelial malignancy exhibiting marked geographic variation (1). Disability-adjusted life-years (DALYs) represent the total healthy life years lost from disease onset to death and are calculated as the sum of years lived with disability and years of life lost due to premature mortality (2). In 2021, an estimated 118,878 new cases were recorded globally across all age groups, resulting in 75,359 deaths and 2.34 million DALYs (3). In middle-aged and older adult populations, endemic regions of East and Southeast Asia accounted for more than 70% of cases, with incidence rates exceeding 3 per 100,000, compared with rates below 0.5 per 100,000 in non-endemic areas (4). Within China alone, 24,000 incident cases were reported in 2021, with rates among men surpassing those of women (5). Among adolescents and young adults, temporal trends from 1990 to 2019 demonstrated a 1.79% average annual increase in incidence and a 2.97% rise in prevalence. In contrast, mortality and DALYs rates declined by 1.64% and 1.60% per year (6). Over the same period, cohorts of middle-aged and older adult individuals experienced a 58.2% increase in incidence (4).

A variety of environmental and behavioral exposures have been implicated in NPC pathogenesis; among these, alcohol consumption has been identified as a modifiable risk factor and one of the major contributors to NPC-related mortality in middle-aged and older adult populations (6). In a comprehensive review of NPC epidemiology, alcohol was found to interact with genetic and environmental determinants in endemic regions, further underscoring its role as a modifiable risk factor (7). A pooled meta-analysis of case-control studies indicated that ever-drinkers had a 1.21-fold increased odds of developing NPC (99% CI, 1.00–1.46) compared with abstainers, with a J-shaped dose-response relationship peaking at more than 15 drinks per week (8). Furthermore, alcohol use has been associated with poorer outcomes; in one retrospective cohort, 5-year overall survival was 70.2% among current drinkers vs. 76.4% among non-drinkers (9). Beyond NPC, alcohol consumption has been linked to both incidence and adverse prognosis across multiple cancer types. In 2020, over 740,000 new cancer cases (~4.1% of all diagnoses) were attributable to alcohol use (10).

Nevertheless, the epidemiology of NPC attributable to alcohol use (NPC-AU) at global, regional, and national levels remains incompletely characterized. Since 1990, the Global Burden of Disease (GBD) study has produced systematic disease burden estimates across 204 countries and territories to inform health policy and practice (11). Recent analyses leveraging GBD 2021 data have begun to clarify trends and preventive strategies for alcohol-related disease burden (12). In this context, NPC-AU was defined as the fraction of NPC mortality and DALYs directly attributable to alcohol use. The present study employed GBD 2021 data to quantify the burden of NPC-AU from 1990 through 2021 and to project trends to 2040. Results were stratified by country, sex, and age to provide evidence supporting targeted prevention and control efforts for NPC-AU.

## Methods

### Data sources and acquisition

Data about NPC-AU were extracted from the GBD 2021 study, which systematically quantifies the burden of 371 diseases and injuries and 88 risk factors across 204 countries and territories between 1990 and 2021. Metrics of interest, including deaths and DALYs, were retrieved through the Global Health Data Exchange platform (http://ghdx.healthdata.org/gbd-results-tool) (11, 13). This study was conducted as a secondary analysis of GBD 2021 data.

### Definitions

In this study, nasopharyngeal carcinoma (NPC) was defined by the 10th revision of the International Statistical Classification of Diseases and Related Health Problems (ICD-10) as codes C11.0–C11.9, encompassing malignant neoplasms of the nasopharynx, including its superior, posterior, lateral, and anterior walls, as well as overlapping and unspecified sites (14).

Alcohol use was identified as a modifiable behavioral risk factor contributing to the burden of NPC. In the Global Burden of Disease (GBD) 2021 study, alcohol consumption was assessed based on self-reported intake levels from population-based surveys. Harmful use was defined as consumption exceeding the theoretical minimum risk exposure level—the level of consumption associated with the lowest overall risk of health loss. The burden of NPC attributable to alcohol use (NPC-AU) was estimated in GBD using a comparative risk assessment framework. First, population-attributable fractions (PAFs) were computed by integrating alcohol exposure data with relative risk estimates from epidemiologic studies. These PAFs were then applied to overall NPC mortality and disability-adjusted life years (DALYs) to derive NPC-AU-specific estimates (15).

The Sociodemographic Index (SDI) was utilized as a composite measure to assess the level of development across countries and territories. The SDI incorporates factors such as average income per person, educational attainment, and total fertility rate among women under 25. Each location was assigned an SDI value ranging from 0 to 1, with higher values indicating greater development. For analytical purposes, countries and territories were categorized into five SDI quintiles: low, low-middle, middle, high-middle, and high (16).

### Statistical analyses

Global, regional and national trends in age-standardized mortality rates (ASMR) and disability-adjusted life-years rates (ASDR) for NPC-AU were evaluated across five SDI quintiles, 21 GBD regions, and 200 countries and territories (Data on NPC-AU were not available for Afghanistan, Yemen, Zambia, and Zimbabwe in the GBD 2021 database due to missing or incomplete country-specific estimates for these nations). Temporal changes from 1990 to 2021 were quantified by calculating the average annual percentage change (AAPC) using log-linear regression models [ln(ASR) = α + β × year + ε]; AAPC was computed as [exp(β) – 1] × 100, with 95% confidence intervals derived from model parameters, where positive and negative values denote increasing and decreasing trends, respectively. Analyses were stratified by sex and age groups of 15–19 years, 20–24 years, 25–29 years, 30–34 years, 35–39 years, 40–44 years, 45–49 years, 50–54 years, 55–59 years, 60–64 years, 65–69 years, 70–74 years, 75–79 years, 80–84 years, 85–89 years, 90–94 years, and 95 years or older. The association between SDI and NPC-AU burden was assessed by comparing disease rates across SDI quintiles (low, low-middle, middle, high-middle, and high) to evaluate disparities attributable to socioeconomic variation. Projections of NPC-AU burden through 2040 were generated using a Bayesian age–period–cohort model, which incorporated age, period, and cohort effects to estimate future ASMR and ASDR with 95% credible intervals. All statistical procedures, data management, and visualizations were performed in R version 4.1.1.

## Results

### Trends of NPC-AU

**Figure 1:**
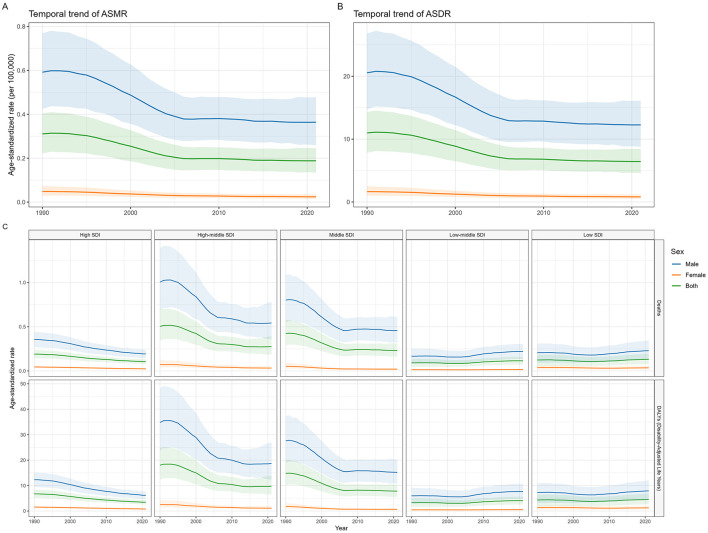
The ASMR and ASDR of NPC-AU from 1990 to 2021 by sex and SDI region. NPC-AU, nasopharynx cancer attributable to alcohol use; ASMR, age-standardized mortality rate; ASDR, age-standardized disability-adjusted life years rate; SDI, Sociodemographic Index.

Between 1990 and 2021, substantial declines were observed in global deaths, DALYs, ASMR, and ASDR for NPC-AU (Figure 1, Table 1). Global ASMR was reduced from 0.31 per 100,000 population [95% uncertainty interval (UI), 0.22–0.40] in 1990 to 0.19 per 100,000 population (95% UI, 0.14–0.24) in 2021, corresponding to AAPC of −1.66 [95% confidence interval (CI), −1.79 to −1.52, *p* < 0.001]. ASDR declined with an AAPC of −1.72 (95% CI, −1.87 to −1.57, *p* < 0.001). From 1990 to 2021, NPC-AU death counts and ASMR were consistently higher in males than in females, with a smaller magnitude of decline; AAPCs were −1.60 (95% CI, −1.75 to −1.46, *p* < 0.001) for males and −2.25 (95% CI, −2.39 to −2.10, p < 0.001) for females.

**Table 1:** Number and age-standardized rates of deaths and DALYs of NPC-AU in 1990 and 2021, with AAPC from 1990 to 2021, stratified by sex, global, 5 SDI regions, and 21 GBD regions.

**Age group**	**Deaths**						**DALYs**					
	**1990**		**2021**		**1990–2021**		**1990**		**2021**		**1990–2021**	
	**Number of deaths cases (95 % UI)**	**Rate of deaths (95 % UI)**	**Number of deaths cases (95 % UI)**	**Rate of deaths (95 % UI)**	**AAPC (95 % CI)**	***P*** **-value**	**Number of DALYs cases (95 % UI)**	**Rate of DALYs (95 % UI)**	**Number of DALYs cases (95 % UI)**	**Rate of DALYs (95 % UI)**	**AAPC (95 % CI)**	***P*** **-value**
15–19	61.79 (37.59 to 89.67)	0.01 (0.01 to 0.02)	47.36 (30.26 to 67.98)	0.01 (0.00 to 0.01)	−1.46 (−1.56 to −1.36)	<0.001	4,530.35 (2,750.90 to 6,565.55)	0.87 (0.53 to 1.26)	3,487.40 (2,226.95 to 4,988.19)	0.56 (0.36 to 0.80)	−1.45 (−1.54 to −1.35)	<0.001
20–24	215.64 (144.55 to 287.02)	0.04 (0.03 to 0.06)	131.40 (91.70 to 174.69)	0.02 (0.02 to 0.03)	−2.24 (−2.48 to −2.01)	<0.001	14,769.69 (9,896.11 to 19,686.56)	3.00 (2.01 to 4.00)	9,092.27 (6,345.34 to 12,036.34)	1.52 (1.06 to 2.02)	−2.21 (−2.45 to −1.98)	<0.001
25–29	275.56 (189.94 to 372.99)	0.06 (0.04 to 0.08)	194.33 (137.93 to 255.80)	0.03 (0.02 to 0.04)	−2.01 (−2.27 to −1.76)	<0.001	17,537.01 (12,067.58 to 23,698.12)	3.96 (2.73 to 5.35)	12,588.58 (8,917.02 to 16,601.58)	2.14 (1.52 to 2.82)	−2.02 (−2.27 to −1.77)	<0.001
30–34	454.69 (317.20 to 614.40)	0.12 (0.08 to 0.16)	402.18 (293.78 to 521.85)	0.07 (0.05 to 0.09)	−1.79 (−2.28 to −1.3)	<0.001	26,596.55 (18,532.91 to 35,910.71)	6.90 (4.81 to 9.32)	24,303.36 (17,715.42 to 31,705.89)	4.02 (2.93 to 5.25)	−1.69 (−2.17 to −1.21)	<0.001
35–39	910.63 (648.18 to 1,202.29)	0.26 (0.18 to 0.34)	696.59 (504.68 to 918.19)	0.12 (0.09 to 0.16)	−2.3 (−2.63 to −1.97)	<0.001	48,738.13 (34,719.90 to 64,490.40)	13.84 (9.86 to 18.31)	38,138.11 (27,606.36 to 50,619.66)	6.80 (4.92 to 9.03)	−2.23 (−2.56 to −1.9)	<0.001
40–44	1,262.50 (907.56 to 1,664.65)	0.44 (0.32 to 0.58)	1,154.80 (839.53 to 1,532.08)	0.23 (0.17 to 0.31)	−2.11 (−2.64 to −1.57)	<0.001	61,305.40 (44,004.97 to 80,861.68)	21.40 (15.36 to 28.23)	56,972.80 (41,340.02 to 75,995.78)	11.39 (8.26 to 15.19)	−2.06 (−2.57 to −1.55)	<0.001
45–49	1,430.69 (1,042.12 to 1,844.80)	0.62 (0.45 to 0.79)	1,663.10 (1,188.53 to 2,195.29)	0.35 (0.25 to 0.46)	−1.84 (−2.12 to −1.57)	<0.001	62,214.48 (45,247.11 to 80,338.63)	26.79 (19.49 to 34.60)	73,280.54 (52,406.03 to 96,985.54)	15.48 (11.07 to 20.48)	−1.8 (−2.08 to −1.53)	<0.001
50–54	1,846.52 (1,369.30 to 2,377.54)	0.87 (0.64 to 1.12)	2,267.81 (1,623.87 to 2,979.06)	0.51 (0.36 to 0.67)	−1.68 (−1.81 to −1.55)	<0.001	71,388.53 (52,911.50 to 91,868.34)	33.58 (24.89 to 43.22)	88,802.67 (63,394.18 to 116,898.36)	19.96 (14.25 to 26.27)	−1.64 (−1.77 to −1.5)	<0.001
55–59	2,054.37 (1,469.77 to 2,670.41)	1.11 (0.79 to 1.44)	2,477.55 (1,816.73 to 3,199.83)	0.63 (0.46 to 0.81)	−1.85 (−1.99 to −1.72)	<0.001	69,733.63 (49,872.47 to 90,716.27)	37.65 (26.93 to 48.98)	84,832.81 (62,175.08 to 109,446.91)	21.44 (15.71 to 27.66)	−1.82 (−1.96 to −1.69)	<0.001
60–64	1,727.44 (1,201.06 to 2,240.84)	1.08 (0.75 to 1.40)	2,073.51 (1,512.92 to 2,649.68)	0.65 (0.47 to 0.83)	−1.64 (−1.89 to −1.39)	<0.001	50,627.50 (35,227.48 to 65,663.26)	31.52 (21.93 to 40.88)	61,169.21 (44,519.86 to 77,913.40)	19.11 (13.91 to 24.34)	−1.62 (−1.87 to −1.38)	<0.001
65–69	1,366.78 (966.80 to 1,745.25)	1.11 (0.78 to 1.41)	2,030.17 (1,435.49 to 2,656.17)	0.74 (0.52 to 0.96)	−1.33 (−1.51 to −1.14)	<0.001	33,763.90 (23,873.41 to 43,079.12)	27.31 (19.31 to 34.85)	50,532.91 (35,712.86 to 66,242.84)	18.32 (12.95 to 24.01)	−1.31 (−1.49 to −1.12)	<0.001
70–74	934.00 (672.60 to 1,210.22)	1.10 (0.79 to 1.43)	1,531.00 (1,092.86 to 1,962.31)	0.74 (0.53 to 0.95)	−1.28 (−1.42 to −1.15)	<0.001	19,009.77 (13,689.32 to 24,633.98)	22.45 (16.17 to 29.10)	31,375.85 (22,438.42 to 40,285.71)	15.24 (10.90 to 19.57)	−1.26 (−1.39 to −1.13)	<0.001
75–79	524.70 (379.79 to 687.49)	0.85 (0.62 to 1.12)	849.77 (595.57 to 1,126.74)	0.64 (0.45 to 0.85)	−0.97 (−1.4 to −0.54)	<0.001	85,93.37 (6,206.89 to 11,264.30)	13.96 (10.08 to 18.30)	14,024.09 (9,827.33 to 18,727.46)	10.63 (7.45 to 14.20)	−0.93 (−1.21 to −0.64)	<0.001
80–84	218.86 (147.67 to 285.25)	0.62 (0.42 to 0.81)	460.36 (311.14 to 616.59)	0.53 (0.36 to 0.70)	−0.55 (−1.07 to −0.03)	0.037	2,802.76 (1,885.48 to 3,659.94)	7.92 (5.33 to 10.35)	5,892.38 (3,978.31 to 7,873.88)	6.73 (4.54 to 8.99)	−0.57 (−0.78 to −0.35)	<0.001
85–89	93.39 (64.58 to 126.19)	0.62 (0.43 to 0.84)	270.58 (176.15 to 364.13)	0.59 (0.39 to 0.80)	−0.23 (−0.72 to 0.27)	0.368	951.65 (656.98 to 1,284.72)	6.30 (4.35 to 8.50)	2,747.64 (1,785.07 to 3,695.92)	6.01 (3.90 to 8.08)	−0.23 (−0.72 to 0.25)	0.344
90–94	22.91 (14.68 to 30.69)	0.53 (0.34 to 0.72)	95.49 (58.68 to 128.43)	0.53 (0.33 to 0.72)	0.03 (−0.24 to 0.31)	0.801	202.54 (129.68 to 271.50)	4.73 (3.03 to 6.34)	844.85 (519.89 to 1,136.35)	4.72 (2.91 to 6.35)	0.04 (−0.23 to 0.31)	0.782
95 plus	3.63 (1.94 to 5.27)	0.36 (0.19 to 0.52)	26.19 (16.16 to 36.14)	0.48 (0.30 to 0.66)	0.95 (0.7 to 1.2)	<0.001	30.23 (16.08 to 43.81)	2.97 (1.58 to 4.30)	214.94 (132.81 to 296.13)	3.94 (2.44 to 5.43)	0.89 (0.71 to 1.08)	<0.001

NPC-AU, nasopharynx cancer attributable to alcohol use; DALYs, disability-adjusted life years; AAPC, average annual percentage change; ASDR, age-standardized DALYs rate; ASMR, age-standardized mortality rate; SDI, sociodemographic Index; CI, confidence interval; UI, uncertainty interval.

ASMR and ASDR decreased across all SDI regions except the low and low-middle SDI groups. Although the high-middle SDI region featured the highest ASMR and ASDR in both 1990 and 2021, it experienced the most pronounced decline: ASMR fell from 0.50 per 100,000 population (95% UI, 0.34–0.69) in 1990 to 0.28 per 100,000 population (95% UI, 0.18–0.39) in 2021 (AAPC, −1.97; 95% CI, −2.11 to −1.83, *p* < 0.001). Notably, the low-middle SDI region saw significant increases in both ASMR and ASDR from 1990 to 2021, with ASMR rising from 0.09 per 100,000 population (95% UI, 0.05–0.14) in 1990 to 0.11 per 100,000 population (95% UI, 0.07–0.16) in 2021 (AAPC, 0.72; 95% CI, 0.51 to 0.92, *p* < 0.001).

**Figure 2:**
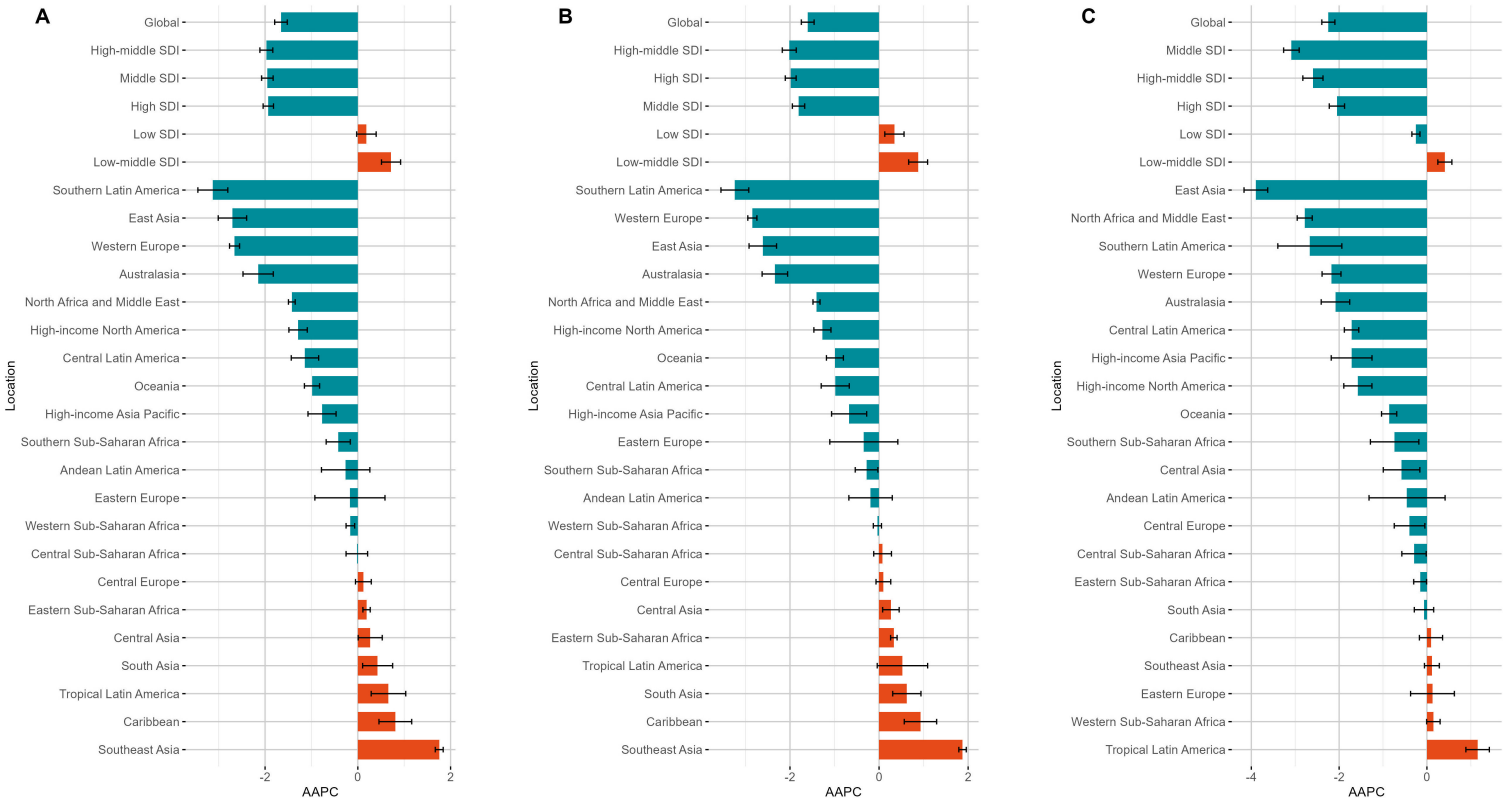
The AAPC of ASMR for NPC-AU at the global level, across the five SDI regions, and within 21 GBD regions, shown for the overall population **(A)**, males **(B)**, and females **(C)**. NPC-AU, nasopharynx cancer attributable to alcohol use; AAPC, average annual percentage change; ASMR, age-standardized mortality rate; SDI, sociodemographic Index.

In GBD regions, East Asia recorded the highest ASMR and ASDR in 1990–0.94 per 100,000 population (95% UI, 0.64–1.28) and 32.52 per 100,000 population (95% UI, 22.26–44.16), respectively—nearly triple the global levels. However, East Asia also ranked second and third in the magnitude of decrease in ASMR and ASDR from 1990 to 2021, with AAPCs of −2.70 (95% CI, −3.01 to −2.40, *p* < 0.001) and −2.70 (95% CI, −2.91 to −2.49, *p* < 0.001). The most significant declines were observed in Southern Latin America [AAPC for ASMR, −3.13 (95% CI, −3.45 to −2.80), *p* < 0.001; AAPC for ASDR, −3.16 (95% CI, −3.43 to −2.88), *p* < 0.001]. Conversely, Southeast Asia exhibited the most pronounced increases [AAPC for ASMR, 1.76 (95% CI, 1.67–1.84, *p* < 0.001); AAPC for ASDR, 1.61 (95% CI, 1.53–1.69), *p* < 0.001]. Notably, in the low SDI regions, male ASMR trended upward from 1990 to 2021 while female ASMR declined [AAPC, 0.34 (95% CI, 0.13–0.56, *p* = 0.002) vs. −0.25 (95% CI, −0.34 to −0.16), *p* < 0.001] (Figure 2, Supplementary Figure S2). Detailed data for global, sex-specific, SDI-specific, and GBD region trends and AAPCs are provided in Table 1 and Supplementary Table S2.

**Figure 3:**
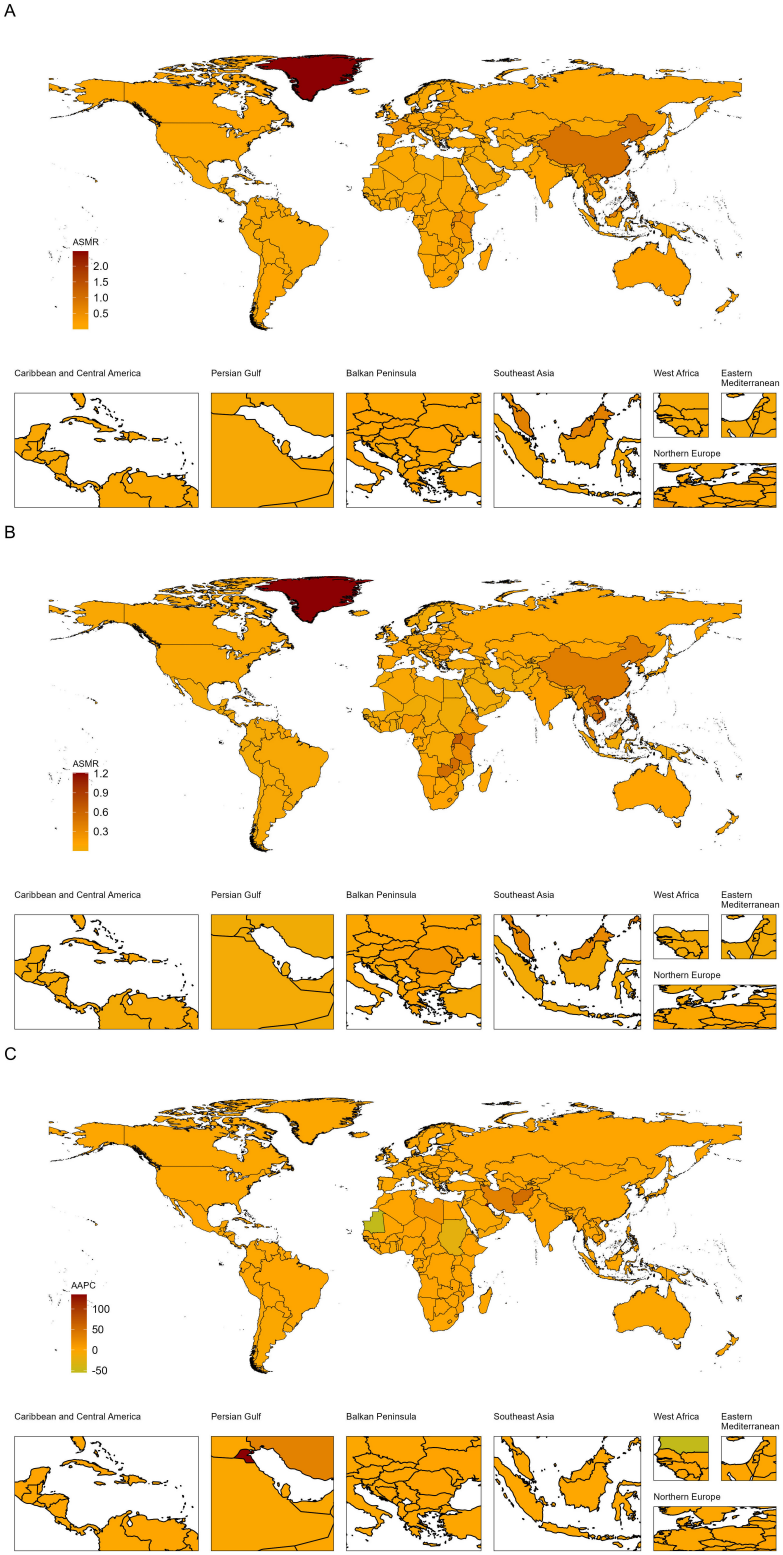
Global ASMR of NPC-AU in 1990 **(A)** and 2021 **(B)**, AAPC **(C)**. AAPC, average annual percentage change; ASMR, age-standardized mortality rate; NPC-AU, nasopharynx cancer attributable to alcohol use.

Among the 200 countries and territories analyzed, Greenland, Taiwan (Province of China), and China exhibited the highest ASMR in 1990–2.47 (95% UI, 1.26–4.10), 1.41 (95% UI, 1.02–1.88), and 0.94 (95% UI, 0.64–1.29) per 100,000 population, respectively. By 2021, the highest values were observed in Greenland, Viet Nam, and Uganda-−1.21 (95% UI, 0.65–1.98), 0.78 (95% UI, 0.41–1.32), and 0.71 (95% UI, 0.36–1.20) per 100,000 population, respectively (Supplementary Table S1). Global maps of ASMR and ASDR for 1990 and 2021 are shown in Figure 3 and Supplementary Figure S3.

### The burden of NPC-AU by age

**Figure 4:**
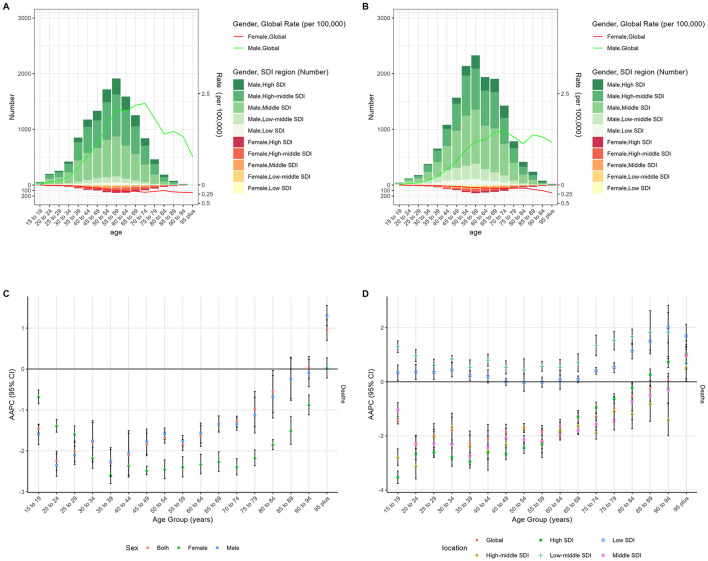
The age distribution of deaths of NPC-AU in different age groups, by sex, in 1990 **(A)** and 2021 **(B)**. AAPC of global ASMR of NPC-AU in different age groups from 1990 to 2021, by sex **(C)** and SDI region **(D)**. NPC-AU, nasopharynx cancer attributable to alcohol use; AAPC, average annual percentage change; ASMR, age-standardized mortality rate; SDI, sociodemographic index; CI, certainty interval.

As shown in Figure 4 and Supplementary Figure S3, ASMR and ASDR for NPC-AU in the overall population increased with age in both 1990 and 2021; in 1990, peaks in ASMR were observed in the 55–59 and 65–69 age groups, whereas in 2021, peaks were observed in the 65–69 and 70–74 age groups. In females, ASMR and ASDR were observed to rise continuously with advancing age, without a distinct peak; in males, both rates peaked in the 70–74 age group in 1990 and 2021. The most pronounced declines in ASMR and ASDR from 1990 to 2021 were observed in the 20–24 years age group, with AAPCs of −2.24 (95% CI, −2.48 to −2.01, *p* < 0.001) and −2.21 (95% CI, −2.45 to −1.98, *p* < 0.001), respectively. Within the 15–90 years age range, increases in ASMR and ASDR were larger in the low-middle SDI regions compared with low SDI regions; this pattern was reversed in the >90 years age group. Numbers and rates of deaths and DALYs of NPC-AU in 1990 and 2021, stratified by age group and accompanied by AAPCs from 1990 to 2021, are presented in Table 2.

**Table 2:** Number and rates of deaths and DALYs of NPC-AU in 1990 and 2021, with AAPC from 1990 to 2021, stratified by age group.

**Age group**	**Deaths**						**DALYs**					
	**1990**		**2021**		**1990–2021**		**1990**		**2021**		**1990–2021**	
	**Number of deaths cases (95 % UI)**	**Rate of deaths (95 % UI)**	**Number of deaths cases (95 % UI)**	**Rate of deaths (95 % UI)**	**AAPC (95 % CI)**	***P*** **-value**	**Number of DALYs cases (95 % UI)**	**Rate of DALYs (95 % UI)**	**Number of DALYs cases (95 % UI)**	**Rate of DALYs (95 % UI)**	**AAPC (95 % CI)**	***P*** **-value**
15–19	61.79 (37.59 to 89.67)	0.01 (0.01 to 0.02)	47.36 (30.26 to 67.98)	0.01 (0.00 to 0.01)	−1.46 (−1.56 to −1.36)	<0.001	4,530.35 (2,750.90 to 6,565.55)	0.87 (0.53 to 1.26)	3,487.40 (2,226.95 to 4,988.19)	0.56 (0.36 to 0.80)	−1.45 (−1.54 to −1.35)	<0.001
20–24	215.64 (144.55 to 287.02)	0.04 (0.03 to 0.06)	131.40 (91.70 to 174.69)	0.02 (0.02 to 0.03)	−2.24 (−2.48 to −2.01)	<0.001	14,769.69 (9,896.11 to 19,686.56)	3.00 (2.01 to 4.00)	9,092.27 (6,345.34 to 12,036.34)	1.52 (1.06 to 2.02)	−2.21 (−2.45 to −1.98)	<0.001
25–29	275.56 (189.94 to 372.99)	0.06 (0.04 to 0.08)	194.33 (137.93 to 255.80)	0.03 (0.02 to 0.04)	−2.01 (−2.27 to −1.76)	<0.001	17,537.01 (12,067.58 to 23,698.12)	3.96 (2.73 to 5.35)	12,588.58 (8,917.02 to 16,601.58)	2.14 (1.52 to 2.82)	−2.02 (−2.27 to −1.77)	<0.001
30–34	454.69 (317.20 to 614.40)	0.12 (0.08 to 0.16)	402.18 (293.78 to 521.85)	0.07 (0.05 to 0.09)	−1.79 (−2.28 to −1.3)	<0.001	26,596.55 (18,532.91 to 35,910.71)	6.90 (4.81 to 9.32)	24,303.36 (17,715.42 to 31,705.89)	4.02 (2.93 to 5.25)	−1.69 (−2.17 to −1.21)	<0.001
35–39	910.63 (648.18 to 1,202.29)	0.26 (0.18 to 0.34)	696.59 (504.68 to 918.19)	0.12 (0.09 to 0.16)	−2.3 (−2.63 to −1.97)	<0.001	48,738.13 (34,719.90 to 64,490.40)	13.84 (9.86 to 18.31)	38,138.11 (27,606.36 to 50,619.66)	6.80 (4.92 to 9.03)	−2.23 (−2.56 to −1.9)	<0.001
40–44	1,262.50 (907.56 to 1,664.65)	0.44 (0.32 to 0.58)	1,154.80 (839.53 to 1,532.08)	0.23 (0.17 to 0.31)	−2.11 (−2.64 to −1.57)	<0.001	61,305.40 (44,004.97 to 80,861.68)	21.40 (15.36 to 28.23)	56,972.80 (41,340.02 to 75,995.78)	11.39 (8.26 to 15.19)	−2.06 (−2.57 to −1.55)	<0.001
45 to 49	1,430.69 (1,042.12 to 1,844.80)	0.62 (0.45 to 0.79)	1,663.10 (1,188.53 to 2,195.29)	0.35 (0.25 to 0.46)	−1.84 (−2.12 to −1.57)	<0.001	62,214.48 (45,247.11 to 80,338.63)	26.79 (19.49 to 34.60)	73,280.54 (52,406.03 to 96,985.54)	15.48 (11.07 to 20.48)	−1.8 (−2.08 to −1.53)	<0.001
50–54	1,846.52 (1,369.30 to 2,377.54)	0.87 (0.64 to 1.12)	2,267.81 (1,623.87 to 2,979.06)	0.51 (0.36 to 0.67)	−1.68 (−1.81 to −1.55)	<0.001	71,388.53 (52,911.50 to 91,868.34)	33.58 (24.89 to 43.22)	88,802.67 (63,394.18 to 116,898.36)	19.96 (14.25 to 26.27)	−1.64 (−1.77 to −1.5)	<0.001
55–59	2,054.37 (1,469.77 to 2,670.41)	1.11 (0.79 to 1.44)	2,477.55 (1,816.73 to 3,199.83)	0.63 (0.46 to 0.81)	−1.85 (−1.99 to −1.72)	<0.001	69,733.63 (49,872.47 to 90,716.27)	37.65 (26.93 to 48.98)	84,832.81 (62,175.08 to 109,446.91)	21.44 (15.71 to 27.66)	−1.82 (−1.96 to −1.69)	<0.001
60–64	1,727.44 (1,201.06 to 2,240.84)	1.08 (0.75 to 1.40)	2,073.51 (1,512.92 to 2,649.68)	0.65 (0.47 to 0.83)	−1.64 (−1.89 to −1.39)	<0.001	50,627.50 (35,227.48 to 65,663.26)	31.52 (21.93 to 40.88)	61,169.21 (44,519.86 to 77,913.40)	19.11 (13.91 to 24.34)	−1.62 (−1.87 to −1.38)	<0.001
65–69	1,366.78 (966.80 to 1,745.25)	1.11 (0.78 to 1.41)	2,030.17 (1,435.49 to 2,656.17)	0.74 (0.52 to 0.96)	−1.33 (−1.51 to −1.14)	<0.001	33,763.90 (23,873.41 to 43,079.12)	27.31 (19.31 to 34.85)	50,532.91 (35,712.86 to 66,242.84)	18.32 (12.95 to 24.01)	−1.31 (−1.49 to −1.12)	<0.001
70–74	934.00 (672.60 to 1,210.22)	1.10 (0.79 to 1.43)	1,531.00 (1,092.86 to 1,962.31)	0.74 (0.53 to 0.95)	−1.28 (−1.42 to −1.15)	<0.001	19,009.77 (13,689.32 to 24,633.98)	22.45 (16.17 to 29.10)	31,375.85 (22,438.42 to 40,285.71)	15.24 (10.90 to 19.57)	−1.26 (−1.39 to −1.13)	<0.001
75–79	524.70 (379.79 to 687.49)	0.85 (0.62 to 1.12)	849.77 (595.57 to 1,126.74)	0.64 (0.45 to 0.85)	−0.97 (−1.4 to −0.54)	<0.001	8,593.37 (6,206.89 to 11,264.30)	13.96 (10.08 to 18.30)	14,024.09 (9,827.33 to 18,727.46)	10.63 (7.45 to 14.20)	−0.93 (−1.21 to −0.64)	<0.001
80–84	218.86 (147.67 to 285.25)	0.62 (0.42 to 0.81)	460.36 (311.14 to 616.59)	0.53 (0.36 to 0.70)	−0.55 (−1.07 to −0.03)	0.037	2,802.76 (1,885.48 to 3,659.94)	7.92 (5.33 to 10.35)	5,892.38 (3,978.31 to 7,873.88)	6.73 (4.54 to 8.99)	−0.57 (−0.78 to −0.35)	<0.001
85–89	93.39 (64.58 to 126.19)	0.62 (0.43 to 0.84)	270.58 (176.15 to 364.13)	0.59 (0.39 to 0.80)	−0.23 (−0.72 to 0.27)	0.368	951.65 (656.98 to 1,284.72)	6.30 (4.35 to 8.50)	2,747.64 (1,785.07 to 3,695.92)	6.01 (3.90 to 8.08)	−0.23 (−0.72 to 0.25)	0.344
90–94	22.91 (14.68 to 30.69)	0.53 (0.34 to 0.72)	95.49 (58.68 to 128.43)	0.53 (0.33 to 0.72)	0.03 (−0.24 to 0.31)	0.801	202.54 (129.68 to 271.50)	4.73 (3.03 to 6.34)	844.85 (519.89 to 1,136.35)	4.72 (2.91 to 6.35)	0.04 (−0.23 to 0.31)	0.782
95 plus	3.63 (1.94 to 5.27)	0.36 (0.19 to 0.52)	26.19 (16.16 to 36.14)	0.48 (0.30 to 0.66)	0.95 (0.7 to 1.2)	<0.001	30.23 (16.08 to 43.81)	2.97 (1.58 to 4.30)	214.94 (132.81 to 296.13)	3.94 (2.44 to 5.43)	0.89 (0.71 to 1.08)	<0.001

NPC-AU, nasopharynx cancer attributable to alcohol use; DALYs, disability-adjusted life years; AAPC, average annual percentage change; CI, confidence interval; UI, uncertainty interval.

### Associations with the SDI

**Figure 5:**
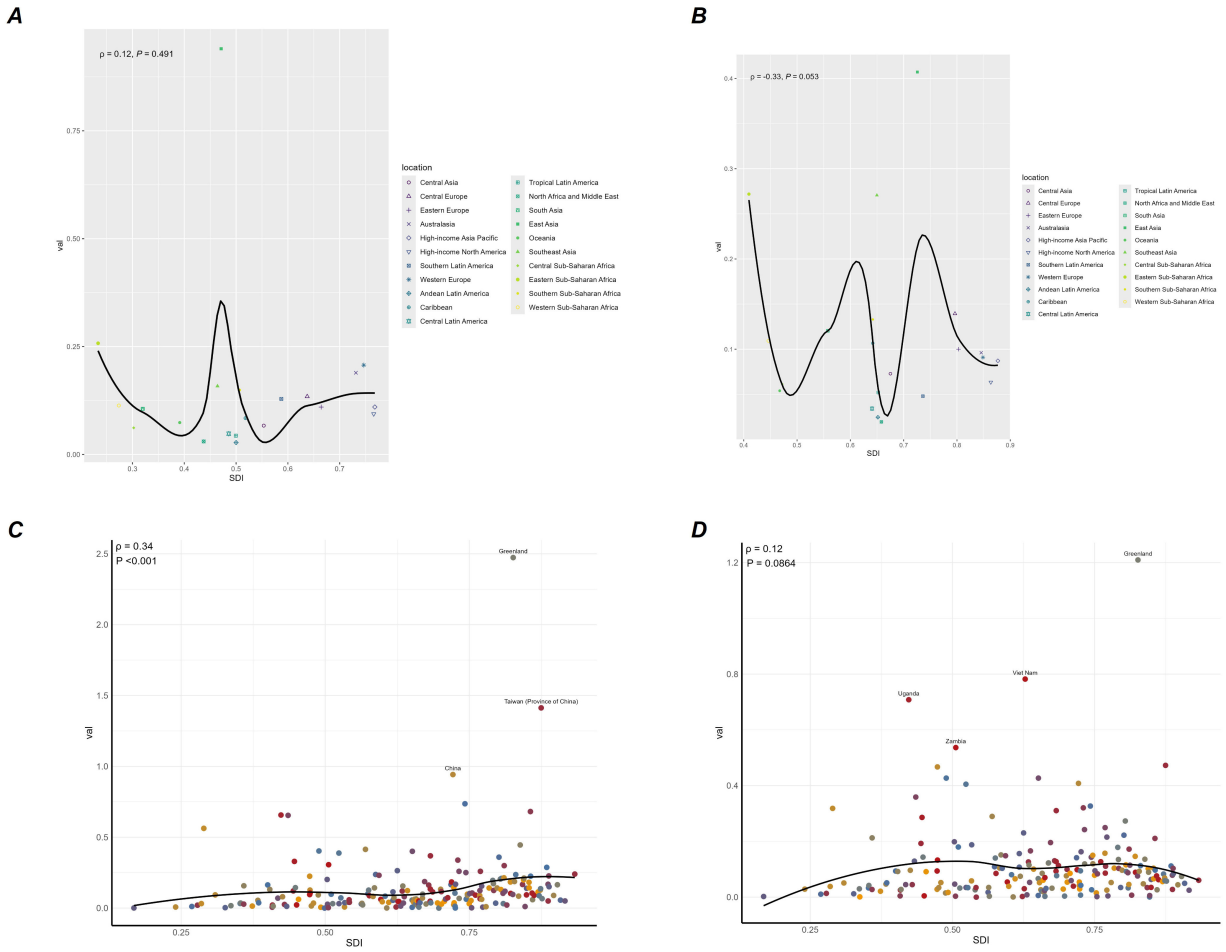
Correlations between ASMR of NPC-AU and SDI at the regional level in 1990 **(A)** and 2021 **(B)**, and at the national level in 1990 **(C)** and 2021 **(D)**. ASMR, age-standardized mortality rate; SDI, socio-demographic Index; NPC-AU, nasopharynx cancer attributable to alcohol use.

Figure 5 and Supplementary Figure S4 illustrate that in 1990, positive correlations were observed between SDI and both ASMR and ASDR at the national level (ρ = 0.34; *p* < 0.001). By 2021, no significant correlations between SDI and ASMR or ASDR were observed at the regional or national levels. In 1990, ASMR and ASDR in Greenland, Taiwan (Province of China), and China were substantially higher than predicted by their respective SDI.

### Global burden predictions for NPC-AU

**Figure 6:**
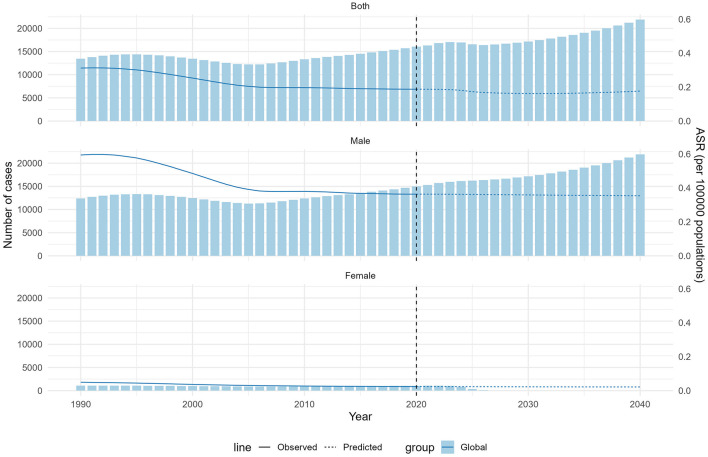
The actual and predicted values in deaths of NPC-AU for the overall population, males, and females. ASR, age-standardized rate; NPC-AU, nasopharynx cancer attributable to alcohol use.

A Bayesian age–period–cohort model was applied to project the NPC-AU burden through 2040 (Figure 6, Supplementary Figure S5). Predicted ASMR and ASDR for males and females were forecasted to continue declining steadily until 2040. By 2040, the predicted male ASMR was 0.35 per 100,000 population (95% UI, 0.03–0.67), and the predicted female ASMR was 0.02 per 100,000 population (95% UI, 0.00–0.04).

## Discussion

This analysis used GBD 2021 data to assess NPC-AU burden from 1990 through 2021 across age, sex, SDI quintiles, and regions, and a BAPC model was applied to project trends through 2040. Temporal trends were evaluated by average annual percentage changes in ASMR and ASDR, revealing that, unlike higher-development settings, low-development regions continue to experience rising NPC-AU burden. Moreover, the age group bearing the most tremendous burden has shifted toward older populations, while the burden among younger cohorts has steadily declined. These findings highlight that, despite global improvements, targeted interventions are urgently needed in low-development areas and for the aging population to mitigate the ongoing impact of alcohol on NPC-AU outcomes.

In this study, marked reductions in alcohol-attributable NPC-AU mortality and disability were observed from 1990 to 2021; declines in overall NPC-AU burden have paralleled these trends reported in recent GBD updates, highlighting a growing incidence amid falling mortality and DALYs (17). Previous analyses have indicated that alcohol use remained one of the leading contributors to NPC-related DALYs, particularly in low SDI regions where declines in mortality have lagged behind those in higher-income settings (18). Furthermore, global cancer burden assessments have attributed a substantial proportion of alcohol-attributable cancer deaths to heavier drinking patterns, reinforcing the need for population-level preventive strategies (10).

Declines in ASMR and ASDR were evident across all SDI strata except for low and low-middle regions, where burdens continued to increase. These findings align with observational analyses of NPC burden from 2009 to 2019, which identified the highest age-standardized rates in high-middle SDI regions but noted the rising age-standardized rates in low-development areas (18). These trends may be partially attributed to advances in cancer treatment technologies that have disproportionately benefited more developed regions (19–22). However, the persistent or rising burden of NPC-AU in low/low-middle SDI regions reflects a confluence of factors specifically impacting alcohol-related cancer control. Disparities in access to timely diagnosis and advanced treatment modalities remain significant in these settings, limiting the ability to effectively manage NPC-AU (23). Crucially, the prevalence and patterns of alcohol consumption exhibit strong socioeconomic gradients. While higher SDI regions have seen more substantial declines in heavy and hazardous drinking prevalence alongside stricter alcohol control policies (24), lower SDI regions often face rapidly evolving alcohol environments characterized by increasing affordability, aggressive marketing, and weaker regulatory enforcement, contributing to rising or sustained high levels of alcohol-attributable risk (25).

A focused analysis of adolescents and young adults with NPC demonstrated stable or increasing incidence in low-SDI zones compared with marked declines in higher SDI settings (6), which may reflect early exposure to these evolving alcohol consumption patterns in transitioning economies. Furthermore, the impact of alcohol on NPC-AU outcomes is amplified in lower-resource settings where comorbid conditions associated with heavy drinking (e.g., malnutrition, liver disease) are more prevalent and access to supportive care is limited. Similar socioeconomic disparities have been documented in head and neck cancers broadly, with mortality declines concentrated in high-income settings while lower-income regions experienced slower progress (26, 27). Regional heterogeneity, such as accelerating declines in Southern Latin America and increasing burdens in Southeast Asia, reflects patterns observed in broader GBD analyses of pharyngeal cancers attributable to alcohol and tobacco (17), underscoring the role of alcohol. Comparable national-level risk assessments have underscored the rising NPC burden in transitional economies, reinforcing the potent link between development level, exposure to key risk factors like alcohol, and the ability to implement effective interventions to reduce the burden of NPC-AU (28).

Findings from this study indicate that the geographic distribution of alcohol-attributable NPC mortality has undergone a notable transition, with regions historically bearing the most tremendous burden—Greenland, Taiwan, and China—giving way to emerging hotspots in Vietnam and Uganda by 2021. Such heterogeneity aligns with GLOBOCAN 2020 data, which reported ASMRs of 1.5 per 100,000 in Eastern Asia and 5.4 per 100,000 in Southeastern Asia compared with rates below 0.2 per 100,000 in Western regions (29). This shifting pattern is further supported by Chen et al. (30), who demonstrated a 1.42-fold increased NPC risk associated with heavy alcohol consumption and a J-shaped dose-response curve in Asian cohorts, and by multifactorial reviews highlighting the interaction of genetic susceptibility, EBV infection, and environmental exposures, including alcohol, in NPC pathogenesis (31). In Vietnam, attributable-cause estimates have suggested that one in ten cancers could be linked to alcohol use, emphasizing its substantial role in the local burden (32). The necessity of implementing alcohol control policies and targeted screening in Greenland and parts of East Asia, South Asia, and Africa is reflected in these data.

NPC-AU's ASMR and ASDR exhibited notable regional divergence. In 1990, East Asia recorded the highest ASMR and ASDR, nearly three times the global averages. Despite these elevated levels, the region experienced considerable declines through 2021. In contrast, Southeast Asia showed the most pronounced increases over the same period. Cultural and religious factors likely help explain these divergent trends. In predominantly Muslim regions, where alcohol consumption is limited by religious prohibition, lower alcohol intake may contribute to reduced NPC-AU mortality and disability (33). Conversely, in parts of East and Southeast Asia, alcohol consumption is deeply embedded in social and ceremonial customs such as family gatherings and festivals (34), which aligns with the observed elevation in ASMR and ASDR. These findings underscore the importance of considering not only healthcare access and treatment advances but also cultural practices, religious norms, and social drinking behaviors when interpreting regional variations in NPC-AU burden.

In this study, NPC-AU mortality was found to rise steadily with age, with peak mortality shifting from middle-aged cohorts in 1990 to older age groups by 2021, and the most pronounced declines were observed among young adults. A population-based investigation in southern China reported that the median age at diagnosis increased from 48 to 56 years over the past three decades, mirroring our findings (1). Unlike the bimodal age peaks described in men, female NPC risk has been shown to climb continuously with advancing age, a pattern attributed to estrogen-mediated protection early in life and sex-specific differences in alcohol metabolism (35, 36). Age-incidence curves from Hong Kong registries similarly demonstrated a continuous rise in female rates after age 50, whereas male rates peaked near age 70 (28). These comparative observations underscore the necessity of age-stratified screening and preventive strategies, particularly for older adults who now bear the most significant burden of NPC-AU (37).

Our analysis suggests globally higher NPC-AU burden among males vs. females during 1990–2021, evidenced by persistently elevated ASMR/ASDR and slower AAPC reductions in males. This disparity may largely reflect differential alcohol exposure patterns, as males typically report greater heavy drinking globally. Such patterns could promote carcinogenic ethanol metabolite accumulation in nasopharyngeal tissues, potentially compounded by gender-based metabolic variations (38). Synergistic interactions with tobacco use—more prevalent among males—might further amplify risk (39). Consequently, male-focused alcohol harm reduction strategies may warrant greater consideration.

Findings from this study demonstrate that the national-level correlation between SDI and NPC-AU burden observed in 1990 had largely dissipated by 2021, and projections indicate that the burden will continue to decline if current trends persist. This attenuation of the development gradient mirrors results from Mahdavifar et al. (40), who found that in 2012 NPC mortality was disproportionately high in moderate-HDI (human development index) countries (ASMR of 1.2 per 100,000 in low HDI vs. 0.7 per 100,000 in high HDI regions) but less divergent in higher-HDI regions. Similarly, a study of mortality-to-incidence ratios showed a strong inverse correlation with health expenditure per capita, indicating that increased investment in health systems mitigates socioeconomic disparities in outcomes (41). Moreover, one review highlighted that as standardized screening and treatment protocols have expanded globally, geographic disparities in NPC outcomes have narrowed (1). The loss of a transparent SDI gradient may thus reflect the diffusion of early detection and evidence-based care across diverse settings. These observations underscore the importance of sustaining equitable resource allocation and universal alcohol-control policies to preserve and accelerate reductions in NPC-AU burden.

Several limitations of this study should be acknowledged. First, it is limited by variable data quality and availability, requiring modeling to fill gaps that may introduce bias, especially in certain regions (42). Second, the projection of future burden was based on historical trends and model assumptions that may not fully capture evolving alcohol consumption patterns or the impact of emerging interventions (43). Third, age-standardized rates may obscure absolute changes in case numbers in populations undergoing rapid demographic transition, potentially underestimating the public health impact in high-growth regions (44).

## Conclusions

Significant declines in ASMR and ASDR for NPC-AU were observed globally from 1990 through 2021. The most significant reductions were achieved in high-middle and middle SDI regions, whereas burdens continued to rise in low and low-middle SDI settings. Male burden remained substantially higher than female burden, and the age at which peak rates occurred shifted upward. Projections indicate that declines in ASMR and ASDR for NPC-AU will continue in both men and women through 2040.

## Data Availability

The original contributions presented in the study are included in the article/Supplementary material, further inquiries can be directed to the corresponding author.
