# Supplementary material for: Global burden of nasopharyngeal carcinoma attributable to alcohol use: a 1990–2021 analysis with projections to 2040
Source: Front Public Health. 2025 Aug 7;13:1623089. doi: 10.3389/fpubh.2025.1623089 (PMC12367759; doi:10.3389/fpubh.2025.1623089)
Supplement: Supplementary file 1 [file Data_Sheet_1.pdf]

Supplementary Materials

Figure S1. The AAPC of ASDR for NPC-AU at the global level, across the five SDI regions, and within 21 GBD regions, shown for the overall population (A), males (B), and females (C). 2

Figure S2. Global ASDR of NPC-AU in 1990 (A) and 2021 (B), AAPC (C). 3

Figure S3. The age distribution of DALYs of NPC-AU in different age groups, by sex, in 1990 （A） and 2021 (B). AAPC of global DALYs rates of NPC-AU in different age groups from 1990 to 2021, by sex (C) and SDI region (D). 4

Figure S4 Correlations between ASDR of NPC-AU and SDI at the regional level in 1990 (A) and 2021 (B), and at the national level in 1990 (C) and 2021 (D). 5

Figure S5. The actual and predicted values in DALYs of NPC-AU for the overall population, males, and females. 6

Table S1. Number and age-standardized rates of deaths and DALYs of NPC-AU in 1990 and 2021, with AAPC from 1990 to 2021, across 204 countries and territories. 7

Table S2. The AAPC of ASDR for NPC-AU at the global level, across the five SDI regions, and within 21 GBD regions, shown for the overall population (A), males (B), and females (C). 19

Table S3. The actual and predicted values in ASMR and number of deaths cases of NPC-AU. .. 21

Table S4. The actual and predicted values in ASDR and number of deaths cases of NPC-AU. ... 23

\

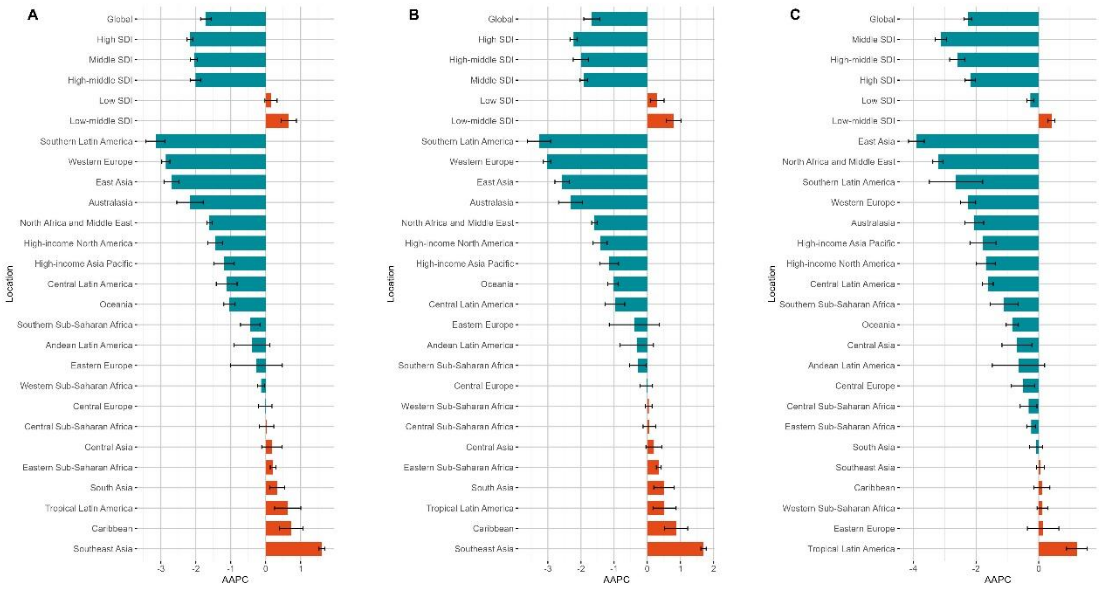

Figure S1. The AAPC of ASDR for NPC-AU at the global level, across the five SDI regions, and within 21 GBD regions, shown for the overall population (A), males (B), and females (C).

Abbreviations: NPC-AU = Nasopharynx cancer attributable to alcohol use; AAPC = average annual percentage change; ASDR = age-standardized disability-adjusted life years rate; SDI = Socio-demographic Index.

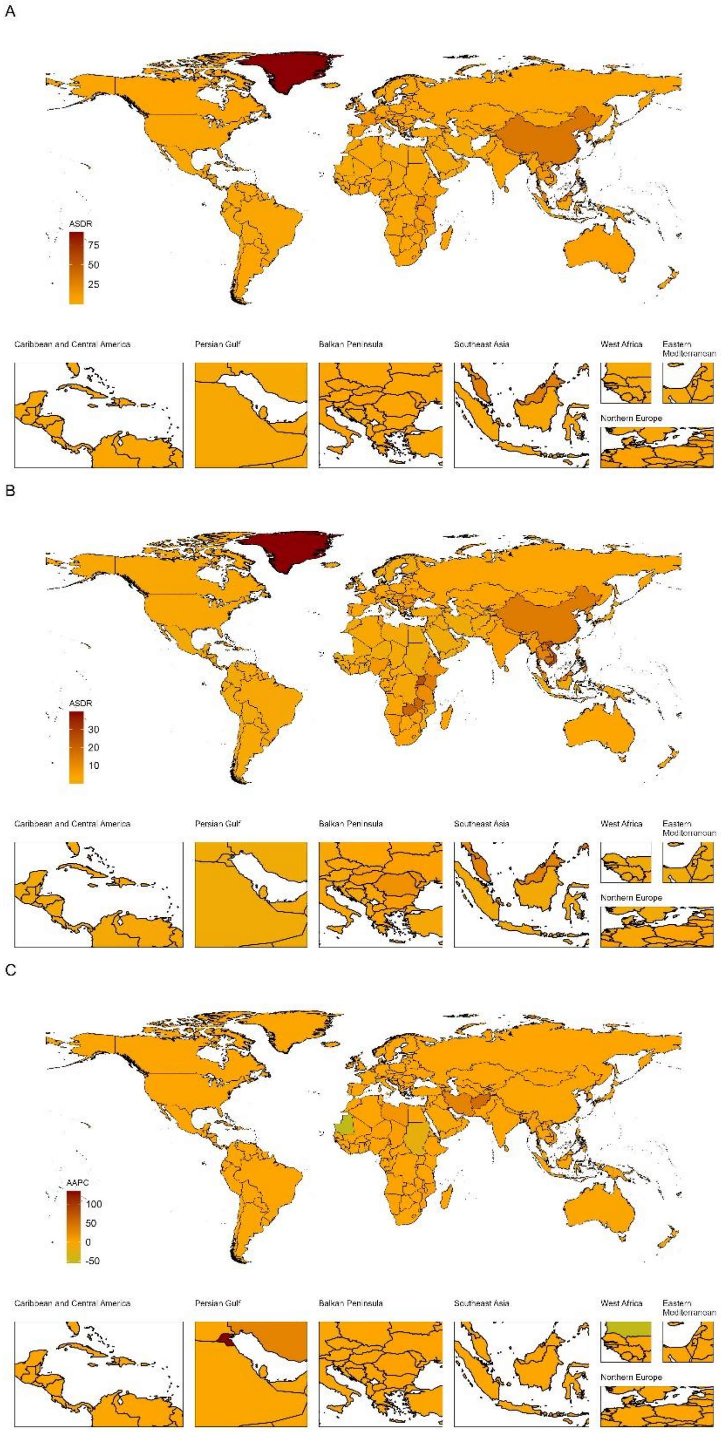

Figure S2. Global ASDR of NPC-AU in 1990 (A) and 2021 (B), AAPC (C).

Abbreviations: AAPC = average annual percentage change; ASDR = age-standardized disability-adjusted life years rate; NPC-AU = Nasopharynx cancer attributable to alcohol use.

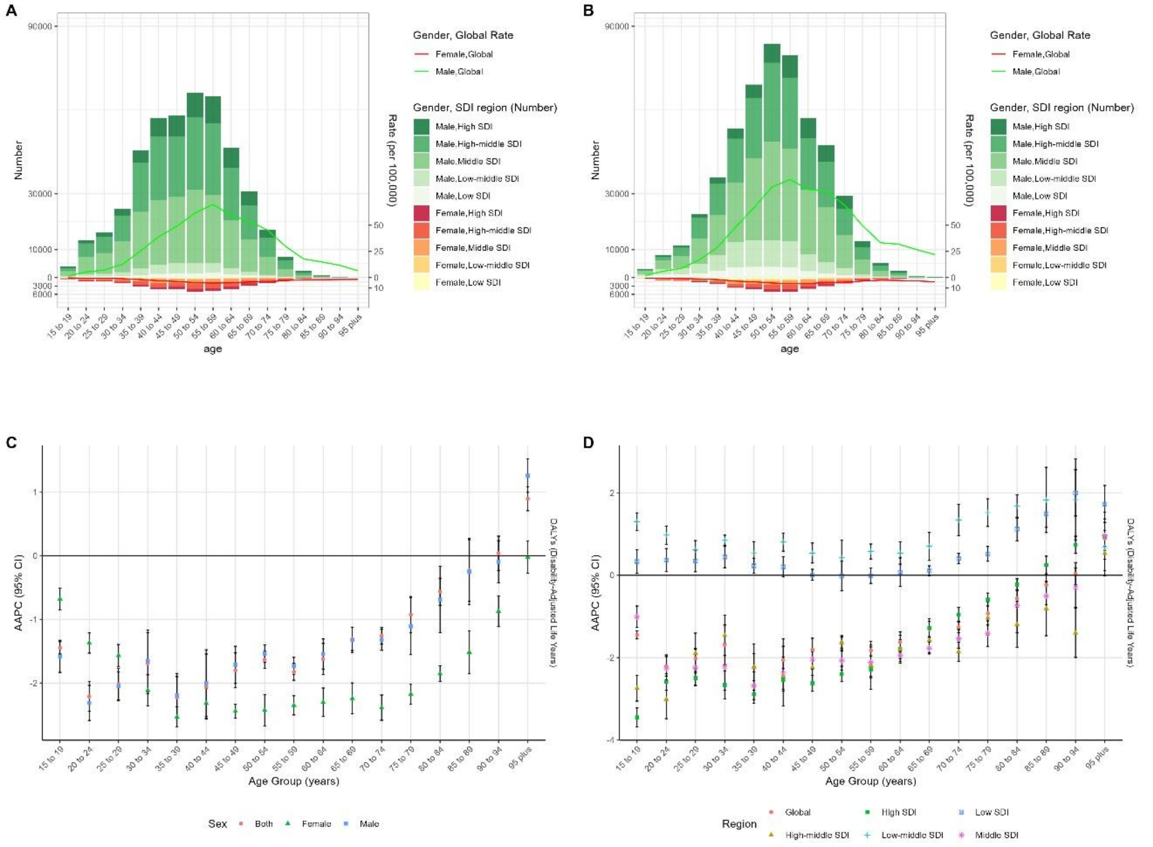

Figure S3. The age distribution of DALYs of NPC-AU in different age groups, by sex, in 1990 （A） and 2021 (B). AAPC of global DALYs rates of NPC-AU in different age groups from 1990 to 2021, by sex (C) and SDI region (D).

Abbreviations: NPC-AU = Nasopharynx cancer attributable to alcohol use; AAPC = average annual percentage change; DALYs = disability-adjusted life years; SDI = Socio-demographic Index; CI = certainty interval.

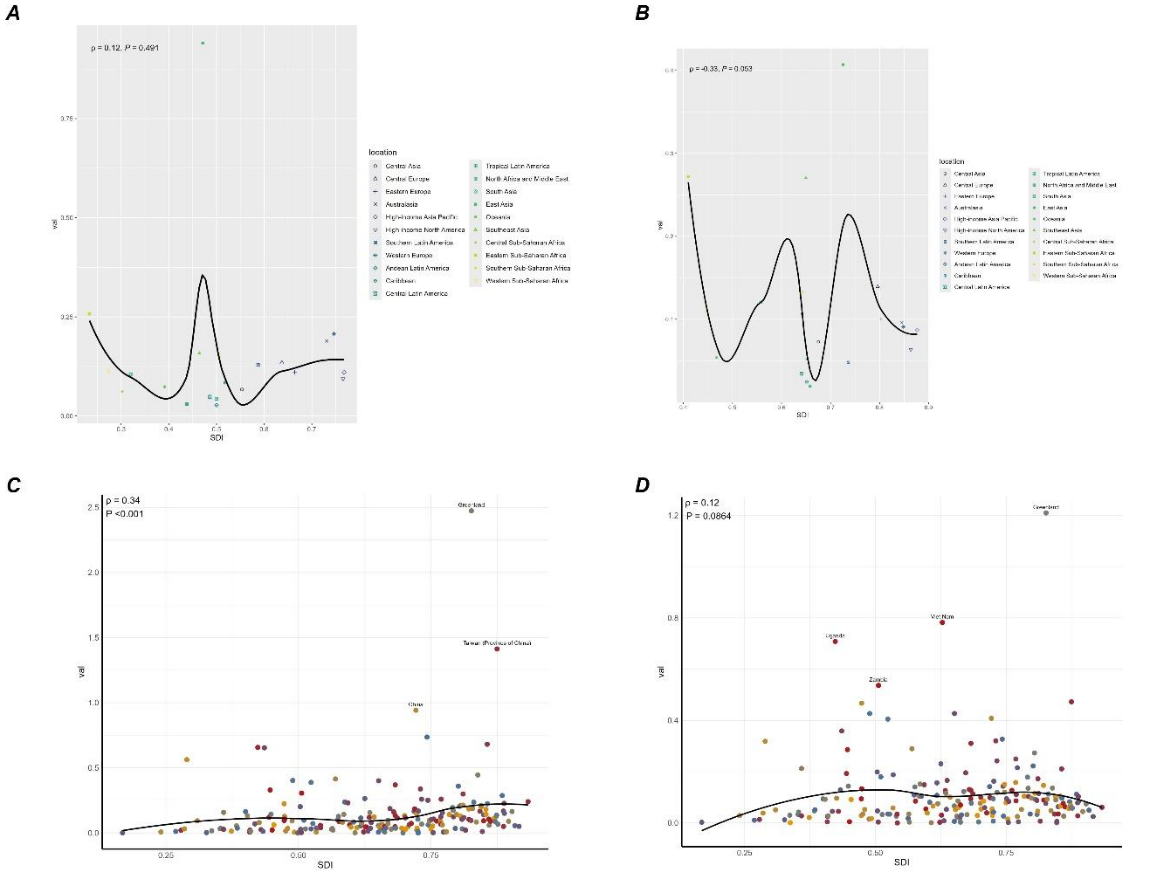

Figure S4 Correlations between ASDR of NPC-AU and SDI at the regional level in 1990 (A) and 2021 (B), and at the national level in 1990 (C) and 2021 (D).

Abbreviations: ASDR = age-standardized disability-adjusted life years rate; SDI=Socio-demographic Index; NPC-AU = Nasopharynx cancer attributable to alcohol use.

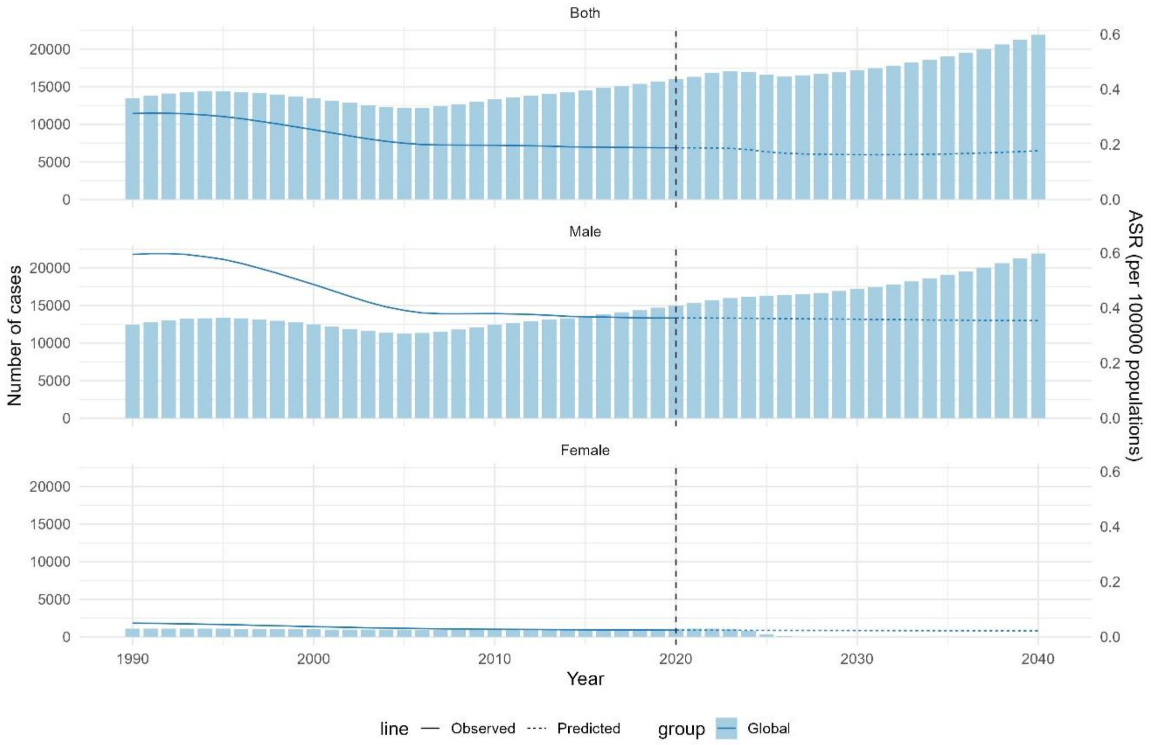

Figure S5. The actual and predicted values in DALYs of NPC-AU for the overall population, males, and females.

Abbreviations: ASR = age-standardized rate; NPC-AU = Nasopharynx cancer attributable to alcohol use; DALYs = disability-adjusted life year

Table S1. Number and age-standardized rates of deaths and DALYs of NPC-AU in 1990 and 2021, with AAPC from 1990 to 2021, across 200 countries and territories.

204 countries and territories	Deaths						DALYs					
	1990		2021		1990-2021		1990		2021		1990-2021	
	Number of deaths cases (95 % UI)	ASMR (95 % UI)	Number of deaths cases (95 % UI)	ASMR (95 % UI)	AAPC (95 % CI)	P-value	Number of DALYs cases (95 % UI)	ASDR (95 % UI)	Number of DALYs cases (95 % UI)	ASDR (95 % UI)	AAPC (95 % CI)	P-value
Albania	1.13（0.47 to 2.04）	0.05（0.02 to 0.09）	3.42（1.77 to 5.74）	0.09（0.04 to 0.14）	1.79 (1.38 to 2.21)	<0.001	41.93（18.12 to 74.92）	1.62（0.69 to 2.91）	104.09（55.07 to 174.15）	2.81（1.48 to 4.72）	1.65 (1.2 to 2.1)	<0.001
Algeria	8.04（3.33 to 15.60）	0.05（0.02 to 0.10）	25.41（10.81 to 49.49）	0.06（0.03 to 0.12）	0.46 (0.35 to 0.58)	<0.001	347.69（147.79 to 670.85）	2.05（0.86 to 3.96）	1013.88（437.54 to 1970.53）	2.30（0.98 to 4.48）	0.36 (0.26 to 0.47)	<0.001
American Samoa	0.01（0.00 to 0.03）	0.04（0.00 to 0.12）	0.02（0.00 to 0.05）	0.03（0.00 to 0.08）	-0.65 (-1.11 to -0.18)	0.007	0.40（0.02 to 1.27）	1.29（0.06 to 4.17）	0.58（0.06 to 1.64）	1.05（0.11 to 2.98）	-0.57 (-1.06 to -0.07)	0.025
Andorra	0.07（0.03 to 0.12）	0.11（0.06 to 0.20）	0.08（0.04 to 0.14）	0.06（0.03 to 0.10）	-2.41 (-2.69 to -2.13)	<0.001	2.26（1.15 to 3.93）	3.76（1.91 to 6.56）	2.61（1.25 to 4.57）	1.85（0.89 to 3.25）	-2.41 (-2.64 to -2.18)	<0.001
Angola	3.10（0.94 to 5.78）	0.06（0.02 to 0.12）	14.09（6.70 to 24.64）	0.09（0.04 to 0.16）	1.29 (1.01 to 1.57)	<0.001	118.40（35.41 to 221.59）	2.10（0.63 to 3.91）	540.61（254.99 to 953.30）	3.06（1.45 to 5.35）	1.22 (0.89 to 1.56)	<0.001
Antigua and Barbuda	0.02（0.01 to 0.03）	0.03（0.01 to 0.05）	0.09（0.06 to 0.12）	0.08（0.05 to 0.11）	3.13 (2.76 to 3.5)	<0.001	0.53（0.23 to 0.89）	1.05（0.46 to 1.77）	2.66（1.77 to 3.69）	2.36（1.57 to 3.29）	2.86 (2.56 to 3.17)	<0.001
Argentina	46.42（32.13 to 62.85）	0.14（0.10 to 0.20）	29.02（18.59 to 42.61）	0.05（0.03 to 0.08）	-3.12 (-3.43 to -2.81)	<0.001	1518.85（1071.74 to 2047.74）	4.70（3.31 to 6.34）	900.98（579.75 to 1319.63）	1.74（1.12 to 2.55）	-3.17 (-3.46 to -2.89)	<0.001
Armenia	0.88（0.45 to 1.33）	0.03（0.01 to 0.04）	2.19（1.26 to 3.24）	0.05（0.03 to 0.08）	2.16 (1.56 to 2.76)	<0.001	34.08（18.21 to 50.67）	1.05（0.55 to 1.57）	72.03（43.95 to 103.85）	1.88（1.17 to 2.70）	1.99 (1.34 to 2.64)	<0.001
Australia	37.69（22.87 to 54.18）	0.20（0.12 to 0.28）	40.10（25.65 to 57.67）	0.10（0.07 to 0.15）	-2.2 (-2.54 to -1.86)	<0.001	1260.21（802.27 to 1770.14）	6.79（4.35 to 9.49）	1220.45（793.19 to 1747.25）	3.46（2.25 to 4.96）	-2.21 (-2.61 to -1.8)	<0.001
Austria	14.77（9.91 to 20.22）	0.14（0.10 to 0.19）	10.35（6.87 to 14.90）	0.07（0.04 to 0.09）	-2.49 (-2.65 to -2.33)	<0.001	469.62（323.52 to 638.27）	4.85（3.36 to 6.58）	294.35（197.25 to 420.80）	2.10（1.41 to 3.01）	-2.7 (-2.93 to -2.48)	<0.001
Azerbaijan	2.10（1.07 to 3.60）	0.04（0.02 to 0.06）	3.38（1.63 to 6.04）	0.03（0.01 to 0.05）	-0.71 (-1.11 to -0.32)	<0.001	83.38（42.80 to 143.10）	1.32（0.68 to 2.27）	123.97（59.72 to 220.63）	1.03（0.49 to 1.83）	-0.84 (-1.31 to -0.37)	0.001
Bahamas	0.23（0.16 to 0.32）	0.13（0.09 to 0.18）	0.51（0.27 to 0.80）	0.12（0.06 to 0.18）	-0.37 (-0.71 to -0.04)	0.029	8.77（6.02 to 12.05）	4.50（3.07 to 6.19）	18.25（9.65 to 28.41）	4.01（2.11 to 6.25）	-0.37 (-0.61 to -0.13)	0.003
Bahrain	0.14（0.07 to 0.26）	0.05（0.02 to 0.10）	0.22（0.09 to 0.43）	0.02（0.01 to 0.03）	-3.92 (-4.16 to -3.68)	<0.001	6.01（2.77 to 11.23）	1.77（0.80 to 3.27）	9.03（3.60 to 17.90）	0.53（0.21 to 1.07）	-3.76 (-4 to -3.52)	<0.001
Bangladesh	3.36（0.00 to 12.26）	0.01（0.00 to	26.14（0.98 to 71.51）	0.02（0.00 to	3.66 (3.44 to	<0.001	139.67（0.00 to 506.08）	0.21（0.00 to	1022.95（41.81 to	0.64（0.03 to	3.64 (3.44 to	<0.001

		0.02)		0.05)	3.87)			0.78)	2830.65)	1.77)	3.84)	
Barbados	0.25 (0.16 to 0.36)	0.10 (0.06 to 0.14)	0.72 (0.45 to 1.06)	0.15 (0.09 to 0.22)	1.63 (0.6 to 2.67)	0.002	8.04 (5.34 to 11.19)	3.24 (2.18 to 4.50)	20.72 (13.01 to 30.27)	4.65 (2.93 to 6.78)	1.26 (0.36 to 2.17)	0.006
Belarus	17.87 (10.65 to 26.04)	0.14 (0.08 to 0.21)	15.66 (8.48 to 24.65)	0.10 (0.06 to 0.16)	-0.95 (-1.47 to -0.43)	<0.001	639.77 (392.05 to 921.82)	5.22 (3.21 to 7.50)	502.91 (282.85 to 776.78)	3.55 (2.03 to 5.45)	-1.21 (-1.75 to -0.67)	<0.001
Belgium	24.77 (16.14 to 34.18)	0.18 (0.12 to 0.25)	20.15 (13.24 to 28.96)	0.10 (0.07 to 0.15)	-1.94 (-2.31 to -1.57)	<0.001	781.05 (524.09 to 1069.12)	6.14 (4.15 to 8.38)	580.92 (383.94 to 831.86)	3.37 (2.22 to 4.83)	-2.07 (-2.47 to -1.66)	<0.001
Belize	0.04 (0.03 to 0.06)	0.04 (0.03 to 0.06)	0.23 (0.15 to 0.33)	0.07 (0.04 to 0.09)	1.61 (1.24 to 1.98)	<0.001	1.49 (0.99 to 2.04)	1.34 (0.89 to 1.85)	8.41 (5.50 to 11.94)	2.23 (1.45 to 3.18)	1.63 (1.27 to 1.98)	<0.001
Benin	0.33 (0.09 to 0.64)	0.01 (0.00 to 0.03)	1.62 (0.65 to 3.16)	0.02 (0.01 to 0.04)	1.4 (1.25 to 1.55)	<0.001	13.86 (3.90 to 26.88)	0.54 (0.15 to 1.04)	71.47 (28.67 to 140.03)	0.86 (0.35 to 1.69)	1.54 (1.39 to 1.69)	<0.001
Bermuda	0.12 (0.07 to 0.17)	0.18 (0.12 to 0.26)	0.18 (0.11 to 0.28)	0.15 (0.09 to 0.23)	-0.82 (-1.09 to -0.55)	<0.001	3.72 (2.42 to 5.27)	5.71 (3.71 to 8.11)	4.86 (2.92 to 7.52)	4.43 (2.68 to 6.88)	-0.87 (-1.08 to -0.66)	<0.001
Bhutan	0.73 (0.27 to 1.38)	0.22 (0.08 to 0.42)	0.20 (0.04 to 0.52)	0.03 (0.01 to 0.07)	-6.53 (-6.81 to -6.24)	<0.001	29.47 (10.92 to 55.62)	8.13 (3.04 to 15.33)	7.97 (1.65 to 20.80)	1.07 (0.22 to 2.80)	-6.39 (-6.69 to -6.09)	<0.001
Bolivia (Plurinational State of)	1.62 (0.73 to 2.91)	0.04 (0.02 to 0.08)	3.78 (1.86 to 6.92)	0.04 (0.02 to 0.07)	-0.44 (-0.53 to -0.35)	<0.001	59.39 (26.28 to 105.95)	1.46 (0.65 to 2.61)	127.69 (61.90 to 235.99)	1.21 (0.59 to 2.24)	-0.6 (-0.67 to -0.53)	<0.001
Bosnia and Herzegovina	1.11 (0.62 to 1.65)	0.02 (0.01 to 0.03)	2.35 (1.45 to 3.38)	0.04 (0.03 to 0.06)	2 (1.62 to 2.38)	<0.001	40.94 (23.31 to 60.61)	0.84 (0.47 to 1.24)	71.86 (44.80 to 103.45)	1.39 (0.86 to 2.01)	1.59 (1.25 to 1.94)	<0.001
Botswana	0.84 (0.36 to 1.55)	0.12 (0.05 to 0.22)	1.84 (0.74 to 3.64)	0.10 (0.04 to 0.19)	-0.64 (-0.78 to -0.49)	<0.001	31.74 (13.61 to 59.80)	4.20 (1.81 to 7.79)	69.91 (27.00 to 141.36)	3.44 (1.37 to 6.83)	-0.62 (-0.75 to -0.5)	<0.001
Brazil	47.77 (33.39 to 63.20)	0.04 (0.03 to 0.06)	133.45 (93.25 to 174.41)	0.05 (0.04 to 0.07)	0.65 (0.27 to 1.03)	0.001	1967.15 (1380.19 to 2593.16)	1.68 (1.18 to 2.22)	5029.43 (3517.74 to 6554.03)	1.98 (1.38 to 2.58)	0.62 (0.24 to 1)	0.001
Brunei Darussalam	0.30 (0.15 to 0.56)	0.21 (0.10 to 0.39)	0.21 (0.02 to 0.46)	0.05 (0.00 to 0.10)	-4.91 (-5.45 to -4.37)	<0.001	12.62 (6.10 to 23.01)	7.31 (3.58 to 13.30)	8.16 (0.86 to 17.91)	1.64 (0.16 to 3.60)	-4.71 (-5.3 to -4.11)	<0.001
Bulgaria	10.28 (6.57 to 14.25)	0.09 (0.06 to 0.12)	18.36 (12.04 to 25.92)	0.16 (0.10 to 0.22)	2.04 (1.33 to 2.76)	<0.001	351.35 (230.12 to 484.22)	3.13 (2.05 to 4.32)	572.83 (380.14 to 805.46)	5.43 (3.61 to 7.64)	1.94 (1.21 to 2.67)	<0.001
Burkina Faso	1.63 (0.73 to 2.81)	0.03 (0.01 to 0.05)	4.85 (2.43 to 8.44)	0.04 (0.02 to 0.07)	0.63 (0.5 to 0.77)	<0.001	64.79 (28.76 to 112.47)	1.18 (0.53 to 2.04)	207.10 (102.33 to 362.46)	1.51 (0.76 to 2.63)	0.81 (0.67 to 0.95)	<0.001
Burundi	15.35 (6.59 to 26.81)	0.56 (0.25 to 0.98)	20.74 (9.31 to 38.47)	0.32 (0.15 to 0.58)	-1.85 (-2.02 to -1.69)	<0.001	583.22 (239.29 to 1026.02)	19.50 (8.33 to 34.16)	819.01 (359.39 to 1537.09)	11.02 (4.96 to 20.42)	-1.85 (-1.95 to -1.75)	<0.001
Cabo Verde	0.02 (0.01 to 0.03)	0.01 (0.00 to 0.01)	0.32 (0.16 to 0.57)	0.06 (0.03 to 0.10)	6.9 (6.68 to 7.12)	<0.001	0.69 (0.29 to 1.24)	0.33 (0.14 to 0.58)	14.00 (6.84 to 25.06)	2.48 (1.22 to 4.42)	6.77 (6.45 to 7.1)	<0.001
Cambodia	7.06 (2.29 to 14.11)	0.13 (0.04 to 0.25)	66.62 (36.36 to 112.24)	0.47 (0.25 to 0.79)	4.31 (4.04 to 4.58)	<0.001	275.07 (89.17 to 550.44)	4.51 (1.47 to 9.01)	2422.16 (1318.89 to 4084.90)	15.88 (8.66 to 26.77)	4.16 (3.92 to 4.41)	<0.001

Cameroon	2.03 （0.83 to 3.53）	0.03 （0.01 to 0.06）	10.03 （4.87 to 17.25）	0.05 （0.03 to 0.09）	1.33 (1.25 to 1.41)	<0.001	84.34 （33.86 to 147.25）	1.35 （0.55 to 2.34）	441.81 （212.95 to 762.05）	2.11 （1.03 to 3.63）	1.48 (1.4 to 1.56)	<0.001
Canada	39.16 （23.59 to 57.32）	0.13 （0.08 to 0.18）	41.84 （27.41 to 60.02）	0.07 （0.05 to 0.10）	-2.02 (-2.39 to -1.65)	<0.001	1362.41 （852.04 to 1940.53）	4.49 （2.82 to 6.38）	1282.14 （859.20 to 1815.60）	2.38 （1.60 to 3.36）	-2.04 (-2.46 to -1.61)	<0.001
Central African Republic	1.31 （0.42 to 2.48）	0.09 （0.03 to 0.18）	1.52 （0.42 to 3.13）	0.05 （0.01 to 0.10）	-1.94 (-2.09 to -1.78)	<0.001	48.55 （15.35 to 92.72）	3.09 （0.99 to 5.89）	59.15 （16.07 to 121.34）	1.74 （0.48 to 3.56）	-1.88 (-2.06 to -1.7)	<0.001
Chad	0.24 （0.03 to 0.64）	0.01 （0.00 to 0.02）	2.50 （0.75 to 5.19）	0.03 （0.01 to 0.06）	4.65 (4.31 to 4.98)	<0.001	10.32 （1.21 to 26.95）	0.29 （0.03 to 0.76）	111.14 （33.47 to 230.75）	1.17 （0.35 to 2.42）	4.69 (4.39 to 4.99)	<0.001
Chile	6.60 （4.32 to 9.32）	0.06 （0.04 to 0.09）	6.21 （4.05 to 9.05）	0.03 （0.02 to 0.04）	-2.78 (-3.19 to -2.37)	<0.001	229.93 （152.59 to 323.14）	2.03 （1.34 to 2.85）	190.82 （126.10 to 276.21）	0.81 （0.53 to 1.17）	-2.93 (-3.57 to -2.28)	<0.001
China	8993.12 （6091.38 to 12287.47）	0.94 （0.64 to 1.29）	8546.60 （5676.94 to 12158.86）	0.41 （0.27 to 0.58）	-2.69 (-2.94 to -2.44)	<0.001	332761.39 （225382.41 to 454707.75）	32.51 （22.03 to 44.40）	285958.74 （191236.33 to 407895.40）	14.14 （9.43 to 20.23）	-2.68 (-2.9 to -2.46)	<0.001
Colombia	13.66 （8.73 to 19.61）	0.06 （0.04 to 0.09）	14.62 （8.14 to 23.58）	0.03 （0.01 to 0.04）	-2.92 (-3.23 to -2.6)	<0.001	562.74 （363.10 to 802.50）	2.25 （1.44 to 3.22）	509.28 （291.47 to 814.37）	0.93 （0.53 to 1.49）	-2.96 (-3.27 to -2.64)	<0.001
Comoros	0.04 （0.00 to 0.12）	0.02 （0.00 to 0.05）	0.20 （0.05 to 0.51）	0.03 （0.01 to 0.09）	2.67 (1.84 to 3.51)	<0.001	1.55 （0.08 to 4.80）	0.55 （0.03 to 1.70）	7.98 （1.80 to 20.38）	1.24 （0.28 to 3.15）	2.65 (1.72 to 3.58)	<0.001
Congo	1.02 （0.28 to 1.89）	0.08 （0.02 to 0.15）	3.54 （1.47 to 6.32）	0.10 （0.04 to 0.18）	0.79 (0.54 to 1.04)	<0.001	37.98 （10.09 to 70.65）	2.75 （0.74 to 5.10）	133.16 （54.59 to 241.09）	3.39 （1.41 to 6.06）	0.69 (0.35 to 1.03)	<0.001
Cook Islands	0.00 （0.00 to 0.00）	0.01 （0.00 to 0.03）	0.01 （0.01 to 0.02）	0.05 （0.03 to 0.09）	5.74 (5.47 to 6)	<0.001	0.05 （0.00 to 0.16）	0.33 （0.00 to 1.06）	0.41 （0.22 to 0.68）	1.71 （0.91 to 2.81）	5.57 (5.3 to 5.85)	<0.001
Costa Rica	2.52 （1.63 to 3.58）	0.13 （0.09 to 0.19）	4.20 （2.49 to 6.49）	0.08 （0.05 to 0.12）	-1.88 (-2.24 to -1.52)	<0.001	88.10 （57.59 to 124.57）	4.24 （2.78 to 6.01）	134.55 （81.24 to 206.93）	2.47 （1.49 to 3.80）	-1.86 (-2.24 to -1.47)	<0.001
Coted'Ivoire	4.45 （1.81 to 8.05）	0.08 （0.03 to 0.14）	15.02 （7.11 to 27.43）	0.10 （0.05 to 0.17）	0.61 (0.44 to 0.77)	<0.001	179.48 （71.56 to 326.86）	2.81 （1.15 to 5.08）	606.76 （283.23 to 1120.20）	3.43 （1.63 to 6.26）	0.65 (0.44 to 0.86)	<0.001
Croatia	8.94 （5.77 to 12.55）	0.14 （0.09 to 0.20）	7.27 （4.45 to 10.44）	0.10 （0.06 to 0.14）	-1.12 (-2.04 to -0.19)	0.019	302.42 （199.20 to 423.34）	4.82 （3.16 to 6.76）	209.23 （131.49 to 298.67）	3.12 （1.97 to 4.46）	-1.29 (-2.22 to -0.36)	0.007
Cuba	7.27 （4.22 to 11.08）	0.07 （0.04 to 0.11）	22.99 （13.89 to 34.99）	0.12 （0.07 to 0.19）	1.94 (1.14 to 2.76)	<0.001	242.92 （147.90 to 362.60）	2.28 （1.38 to 3.41）	683.11 （419.24 to 1030.37）	3.83 （2.35 to 5.81）	1.83 (1.04 to 2.62)	<0.001
Cyprus	0.65 （0.35 to 1.06）	0.08 （0.04 to 0.14）	0.93 （0.53 to 1.49）	0.05 （0.03 to 0.08）	-1.82 (-2.11 to -1.52)	<0.001	20.09 （11.09 to 32.46）	2.51 （1.38 to 4.06）	27.88 （15.69 to 44.81）	1.50 （0.84 to 2.42）	-1.67 (-1.89 to -1.45)	<0.001
Czechia	24.21 （16.23 to 33.15）	0.19 （0.13 to 0.26）	16.87 （10.33 to 24.37）	0.09 （0.06 to 0.13）	-2.21 (-2.41 to -2.01)	<0.001	798.89 （548.95 to 1085.59）	6.39 （4.42 to 8.67）	495.21 （308.07 to 714.31）	3.03 （1.89 to 4.38）	-2.43 (-2.58 to -2.27)	<0.001
Democratic People's Republic of Korea	80.05 （39.61 to 144.00）	0.41 （0.21 to 0.74）	100.31 （50.21 to 178.24）	0.29 （0.14 to 0.52）	-1.15 (-1.21 to -1.1)	<0.001	3078.55 （1506.87 to 5591.36）	15.22 （7.45 to 27.59）	3624.03 （1799.78 to 6509.21）	10.49 （5.20 to 18.94）	-1.2 (-1.27 to -1.14)	<0.001
Democratic Republic of the Congo	10.11	0.05	21.90	0.05	-0.5 (-0.82 to -	0.002	370.63	1.77	829.62	1.56	-0.45 (-0.81 to -	0.016

	（2.66 to 19.85）	（0.01 to 0.11）	（6.54 to 43.68）	（0.01 to 0.09）	0.19)		（95.56 to 729.23）	（0.46 to 3.49）	（244.55 to 1661.62）	（0.46 to 3.11）	0.08)	
Denmark	6.31（4.09 to 8.61）	0.09（0.06 to 0.12）	4.58（2.87 to 6.78）	0.04（0.03 to 0.07）	-2.18 (-2.48 to -1.88)	<0.001	193.68（129.86 to 261.98）	2.93（1.98 to 3.95）	125.17（79.40 to 184.47）	1.37（0.87 to 2.02）	-2.37 (-2.69 to -2.05)	<0.001
Djibouti	0.19（0.02 to 0.46）	0.09（0.01 to 0.22）	0.15（0.01 to 0.42）	0.02（0.00 to 0.04）	-5.7 (-6.04 to -5.37)	<0.001	7.70（0.79 to 19.21）	3.31（0.40 to 8.11）	6.31（0.55 to 17.96）	0.58（0.06 to 1.62）	-5.51 (-5.81 to -5.21)	<0.001
Dominica	0.05（0.03 to 0.08）	0.09（0.05 to 0.14）	0.10（0.05 to 0.17）	0.12（0.06 to 0.20）	0.92 (0.74 to 1.1)	<0.001	1.64（0.96 to 2.56）	2.81（1.66 to 4.39）	3.24（1.73 to 5.44）	3.86（2.05 to 6.49）	1.03 (0.82 to 1.24)	<0.001
Dominican Republic	3.25（1.79 to 5.21）	0.07（0.04 to 0.12）	7.88（4.02 to 13.84）	0.07（0.04 to 0.13）	0.13 (-0.32 to 0.58)	0.579	131.36（75.32 to 207.11）	2.58（1.45 to 4.10）	282.89（147.73 to 491.94）	2.60（1.35 to 4.53）	0.08 (-0.37 to 0.54)	0.719
Ecuador	1.00（0.50 to 1.56）	0.02（0.01 to 0.02）	2.99（1.67 to 4.81）	0.02（0.01 to 0.03）	0.26 (-0.45 to 0.97)	0.478	40.08（19.96 to 62.08）	0.57（0.29 to 0.88）	105.14（59.68 to 167.31）	0.60（0.34 to 0.96）	0.08 (-0.62 to 0.79)	0.817
Egypt	0.28（0.11 to 0.55）	0.00（0.00 to 0.00）	0.66（0.26 to 1.30）	0.00（0.00 to 0.00）	0.12 (-0.41 to 0.66)	0.655	11.84（4.93 to 23.21）	0.03（0.01 to 0.06）	26.44（10.79 to 51.93）	0.03（0.01 to 0.06）	-0.03 (-0.55 to 0.49)	0.914
El Salvador	1.12（0.67 to 1.68）	0.03（0.02 to 0.05）	2.13（1.22 to 3.35）	0.03（0.02 to 0.05）	0.09 (-0.58 to 0.76)	0.799	43.59（26.31 to 64.54）	1.18（0.71 to 1.76）	76.44（44.55 to 119.02）	1.23（0.72 to 1.92）	0.15 (-0.57 to 0.88)	0.678
Equatorial Guinea	0.15（0.04 to 0.30）	0.06（0.02 to 0.13）	0.57（0.23 to 1.06）	0.09（0.04 to 0.16）	0.94 (0.6 to 1.27)	<0.001	5.35（1.33 to 10.97）	2.14（0.54 to 4.38）	22.35（8.73 to 42.65）	2.76（1.11 to 5.10）	0.88 (0.61 to 1.16)	<0.001
Eritrea	2.28（0.36 to 4.61）	0.13（0.02 to 0.27）	3.89（0.94 to 8.57）	0.09（0.02 to 0.20）	-1.18 (-1.3 to -1.06)	<0.001	94.64（14.32 to 192.53）	4.86（0.79 to 9.86）	166.22（38.55 to 365.81）	3.50（0.85 to 7.70）	-1.13 (-1.32 to -0.94)	<0.001
Estonia	4.11（2.27 to 6.16）	0.21（0.12 to 0.32）	1.89（1.06 to 3.00）	0.09（0.05 to 0.14）	-2.83 (-3.53 to -2.13)	<0.001	148.67（86.38 to 219.40）	7.91（4.65 to 11.65）	57.65（33.80 to 88.74）	3.02（1.81 to 4.59）	-3.19 (-3.96 to -2.42)	<0.001
Eswatini	0.48（0.23 to 0.82）	0.14（0.07 to 0.24）	1.05（0.48 to 1.89）	0.15（0.07 to 0.27）	0.19 (0.02 to 0.36)	0.024	17.10（8.03 to 29.74）	4.52（2.15 to 7.74）	39.67（17.54 to 72.41）	5.10（2.31 to 9.19）	0.41 (0.23 to 0.6)	<0.001
Ethiopia	40.56（7.55 to 83.28）	0.16（0.03 to 0.32）	119.22（55.00 to 222.39）	0.21（0.10 to 0.40）	1.01 (0.91 to 1.1)	<0.001	1613.86（275.36 to 3307.93）	5.68（1.03 to 11.67）	4746.79（2147.35 to 8848.58）	7.37（3.41 to 13.75）	0.86 (0.78 to 0.95)	<0.001
Fiji	0.13（0.05 to 0.25）	0.03（0.01 to 0.05）	0.29（0.13 to 0.53）	0.03（0.01 to 0.06）	0.37 (-0.07 to 0.83)	0.102	5.02（1.88 to 9.39）	0.99（0.37 to 1.86）	10.20（4.48 to 18.68）	1.10（0.48 to 2.01）	0.37 (-0.06 to 0.79)	0.089
Finland	3.85（2.41 to 5.37）	0.06（0.04 to 0.08）	2.54（1.58 to 3.79）	0.03（0.02 to 0.04）	-2.56 (-2.75 to -2.36)	<0.001	130.05（84.20 to 179.32）	2.09（1.36 to 2.87）	73.33（46.35 to 108.87）	0.90（0.57 to 1.33）	-2.65 (-2.79 to -2.51)	<0.001
France	317.64（213.60 to 420.80）	0.45（0.30 to 0.59）	153.06（97.75 to 223.06）	0.14（0.09 to 0.20）	-3.78 (-4.04 to -3.51)	<0.001	10437.44（7183.15 to 13765.05）	15.36（10.63 to 20.24）	4404.17（2817.98 to 6408.30）	4.52（2.89 to 6.57）	-3.9 (-4.06 to -3.74)	<0.001
Gabon	0.94（0.44 to 1.56）	0.16（0.07 to 0.26）	1.40（0.67 to 2.43）	0.12（0.06 to 0.20）	-0.95 (-1.09 to -0.82)	<0.001	31.88（14.65 to 53.37）	4.99（2.30 to 8.31）	49.67（23.16 to 87.05）	3.68（1.74 to 6.39）	-0.97 (-1.15 to -0.8)	<0.001
Gambia	0.06（0.02 to 0.12）	0.01（0.00 to 0.03）	0.35（0.16 to 0.64）	0.03（0.01 to 0.05）	2.42 (1.76 to 3.09)	<0.001	2.43（0.60 to 4.86）	0.48（0.12 to 0.95）	14.25（6.36 to 25.92）	1.00（0.45 to 1.81）	2.49 (1.83 to 3.15)	<0.001

Georgia	4.29 (2.05 to 7.23)	0.07 (0.03 to 0.11)	6.20 (3.45 to 9.68)	0.12 (0.07 to 0.18)	1.91 (0.61 to 3.23)	0.004	157.62 (78.23 to 259.32)	2.55 (1.27 to 4.19)	201.18 (117.21 to 306.90)	4.25 (2.52 to 6.43)	1.65 (0.67 to 2.63)	0.001
Germany	184.41 (128.90 to 243.83)	0.16 (0.12 to 0.22)	120.43 (80.76 to 166.91)	0.07 (0.05 to 0.10)	-2.63 (-2.94 to -2.33)	<0.001	6134.20 (4390.99 to 8073.51)	5.78 (4.15 to 7.61)	3275.75 (2212.18 to 4529.34)	2.32 (1.56 to 3.22)	-3 (-3.29 to -2.71)	<0.001
Ghana	2.07 (0.80 to 3.71)	0.02 (0.01 to 0.04)	0.83 (0.39 to 1.54)	0.00 (0.00 to 0.01)	-5.96 (-6.39 to -5.52)	<0.001	93.20 (35.96 to 168.23)	0.96 (0.37 to 1.72)	37.64 (17.53 to 70.29)	0.14 (0.07 to 0.26)	-5.95 (-6.37 to -5.52)	<0.001
Greece	24.51 (16.54 to 32.07)	0.17 (0.12 to 0.23)	25.20 (17.70 to 33.33)	0.14 (0.10 to 0.18)	-0.79 (-1.07 to -0.5)	<0.001	771.07 (539.63 to 999.35)	5.75 (4.06 to 7.44)	717.94 (510.35 to 944.65)	4.42 (3.15 to 5.81)	-0.85 (-1.17 to -0.54)	<0.001
Greenland	1.17 (0.62 to 1.90)	2.47 (1.26 to 4.10)	0.92 (0.50 to 1.50)	1.21 (0.65 to 1.98)	-2.28 (-2.55 to -2)	<0.001	47.60 (25.49 to 76.58)	91.33 (48.00 to 148.44)	30.33 (16.45 to 49.32)	39.75 (21.42 to 64.94)	-2.66 (-2.93 to -2.38)	<0.001
Grenada	0.07 (0.04 to 0.10)	0.10 (0.06 to 0.15)	0.15 (0.09 to 0.22)	0.12 (0.07 to 0.19)	0.79 (0.26 to 1.31)	0.003	2.21 (1.34 to 3.35)	3.37 (2.06 to 5.09)	4.84 (2.98 to 7.23)	3.95 (2.43 to 5.92)	0.55 (0.24 to 0.86)	0.001
Guam	0.20 (0.00 to 0.51)	0.20 (0.00 to 0.54)	0.56 (0.13 to 1.02)	0.27 (0.06 to 0.50)	0.96 (0.37 to 1.55)	0.001	7.20 (0.09 to 18.34)	6.93 (0.08 to 17.86)	19.33 (4.67 to 35.10)	9.81 (2.38 to 17.73)	1.11 (0.47 to 1.75)	0.001
Guatemala	1.71 (1.08 to 2.45)	0.04 (0.03 to 0.06)	3.23 (1.92 to 4.88)	0.03 (0.02 to 0.04)	-1.54 (-2.38 to -0.7)	<0.001	67.10 (42.97 to 95.22)	1.39 (0.88 to 1.98)	121.95 (74.14 to 181.08)	0.92 (0.55 to 1.37)	-1.4 (-2.26 to -0.54)	0.002
Guinea	0.61 (0.12 to 1.28)	0.02 (0.00 to 0.04)	2.08 (0.77 to 4.16)	0.03 (0.01 to 0.06)	1.92 (1.64 to 2.2)	<0.001	22.59 (4.23 to 47.67)	0.59 (0.11 to 1.25)	81.95 (30.31 to 164.66)	1.07 (0.40 to 2.13)	1.94 (1.64 to 2.23)	<0.001
Guinea-Bissau	0.18 (0.08 to 0.34)	0.04 (0.01 to 0.07)	0.50 (0.24 to 0.90)	0.04 (0.02 to 0.07)	0.56 (0.46 to 0.66)	<0.001	7.71 (3.11 to 14.29)	1.37 (0.56 to 2.54)	22.76 (10.77 to 40.68)	1.73 (0.83 to 3.06)	0.78 (0.66 to 0.9)	<0.001
Guyana	0.25 (0.16 to 0.36)	0.06 (0.04 to 0.08)	0.48 (0.26 to 0.79)	0.07 (0.04 to 0.11)	0.55 (0.01 to 1.1)	0.047	9.26 (5.92 to 13.41)	1.88 (1.19 to 2.73)	17.57 (9.73 to 28.68)	2.32 (1.28 to 3.80)	0.72 (0.2 to 1.24)	0.007
Haiti	5.33 (2.57 to 9.26)	0.14 (0.07 to 0.25)	12.00 (5.77 to 21.39)	0.14 (0.07 to 0.26)	0.06 (-0.12 to 0.24)	0.512	193.25 (92.90 to 333.04)	4.70 (2.27 to 8.14)	437.34 (208.86 to 779.56)	4.56 (2.19 to 8.14)	0 (-0.18 to 0.19)	0.973
Honduras	0.73 (0.38 to 1.24)	0.03 (0.02 to 0.05)	2.24 (1.07 to 4.14)	0.03 (0.02 to 0.06)	0.19 (0.05 to 0.32)	0.006	27.24 (14.20 to 46.03)	1.02 (0.53 to 1.73)	74.90 (34.87 to 140.26)	0.99 (0.47 to 1.84)	-0.1 (-0.22 to 0.02)	0.1
Hungary	26.35 (17.43 to 36.28)	0.20 (0.13 to 0.27)	28.63 (18.35 to 40.57)	0.18 (0.12 to 0.25)	-0.29 (-0.73 to 0.16)	0.206	918.02 (622.81 to 1253.91)	7.07 (4.82 to 9.66)	885.88 (576.12 to 1244.72)	5.99 (3.90 to 8.41)	-0.56 (-1.02 to -0.09)	0.018
Iceland	0.18 (0.10 to 0.27)	0.07 (0.04 to 0.10)	0.29 (0.19 to 0.42)	0.06 (0.04 to 0.08)	-0.42 (-0.62 to -0.22)	<0.001	5.88 (3.35 to 8.87)	2.29 (1.32 to 3.44)	8.99 (5.80 to 12.87)	1.93 (1.25 to 2.77)	-0.5 (-0.75 to -0.24)	<0.001
India	736.73 (270.08 to 1174.08)	0.13 (0.05 to 0.20)	1862.84 (1045.13 to 2712.21)	0.14 (0.08 to 0.20)	0.37 (0.16 to 0.58)	0.001	28683.98 (10470.70 to 45455.35)	4.58 (1.68 to 7.28)	68450.37 (39011.75 to 99451.09)	4.95 (2.81 to 7.20)	0.27 (0.08 to 0.47)	0.007
Indonesia	34.75 (10.04 to 71.89)	0.03 (0.01 to 0.06)	62.78 (15.59 to 145.56)	0.02 (0.01 to 0.05)	-0.74 (-0.97 to -0.51)	<0.001	1435.69 (420.34 to 2956.81)	1.02 (0.30 to 2.11)	2419.51 (603.86 to 5669.96)	0.79 (0.20 to 1.84)	-0.83 (-1.03 to -0.63)	<0.001
Iran (Islamic Republic of)	0.00	0.00	3.38	0.00	32.92 (30.36 to	<0.001	0.02	0.00	132.73	0.14	30.95 (28.66 to	<0.001

	(0.00 to 0.00)	(0.00 to 0.00)	(1.72 to 5.45)	(0.00 to 0.01)	35.53)		(0.00 to 0.12)	(0.00 to 0.00)	(70.06 to 212.01)	(0.07 to 0.23)	33.28)	
Iraq	0.66 (0.31 to 1.20)	0.01 (0.00 to 0.01)	0.90 (0.39 to 1.69)	0.00 (0.00 to 0.01)	-2.74 (-3.04 to -2.44)	<0.001	27.98 (13.15 to 50.90)	0.25 (0.12 to 0.46)	36.51 (16.24 to 68.96)	0.10 (0.05 to 0.20)	-2.84 (-3.11 to -2.56)	<0.001
Ireland	5.21 (3.24 to 7.46)	0.14 (0.09 to 0.20)	4.21 (2.64 to 6.06)	0.06 (0.04 to 0.08)	-2.72 (-3 to -2.44)	<0.001	164.30 (106.34 to 232.21)	4.47 (2.92 to 6.30)	130.45 (83.08 to 186.95)	1.95 (1.25 to 2.80)	-2.66 (-2.98 to -2.34)	<0.001
Israel	1.42 (0.53 to 2.60)	0.03 (0.01 to 0.06)	3.61 (1.88 to 5.87)	0.03 (0.02 to 0.05)	0.14 (-0.37 to 0.65)	0.599	50.51 (20.47 to 90.97)	1.13 (0.46 to 2.02)	116.42 (60.92 to 188.60)	1.13 (0.59 to 1.82)	-0.03 (-0.5 to 0.43)	0.883
Italy	167.31 (119.20 to 209.02)	0.21 (0.15 to 0.26)	108.05 (77.16 to 139.64)	0.09 (0.07 to 0.12)	-2.64 (-2.91 to -2.36)	<0.001	5329.18 (3900.08 to 6620.03)	7.11 (5.25 to 8.83)	2981.10 (2155.64 to 3846.90)	2.96 (2.14 to 3.83)	-2.76 (-3 to -2.52)	<0.001
Jamaica	0.64 (0.34 to 1.03)	0.04 (0.02 to 0.06)	2.39 (1.25 to 4.08)	0.08 (0.04 to 0.13)	3.06 (2.1 to 4.02)	<0.001	20.30 (11.25 to 31.82)	1.16 (0.65 to 1.82)	75.24 (39.78 to 127.65)	2.43 (1.28 to 4.12)	2.98 (2 to 3.98)	<0.001
Japan	147.33 (109.33 to 186.00)	0.09 (0.06 to 0.11)	264.58 (182.48 to 344.47)	0.08 (0.06 to 0.11)	-0.19 (-0.69 to 0.32)	0.466	4593.16 (3447.90 to 5769.08)	2.77 (2.08 to 3.48)	5703.45 (4029.68 to 7356.04)	2.31 (1.65 to 2.97)	-0.59 (-1.03 to -0.15)	0.008
Jordan	0.22 (0.09 to 0.45)	0.01 (0.01 to 0.03)	0.70 (0.28 to 1.41)	0.01 (0.00 to 0.01)	-1.73 (-1.97 to -1.49)	<0.001	9.03 (3.72 to 17.81)	0.44 (0.18 to 0.87)	27.28 (11.08 to 55.09)	0.25 (0.10 to 0.51)	-1.73 (-1.97 to -1.48)	<0.001
Kazakhstan	15.01 (9.38 to 21.76)	0.11 (0.06 to 0.15)	18.61 (11.16 to 27.34)	0.09 (0.06 to 0.14)	-0.36 (-0.93 to 0.23)	0.23	593.22 (382.73 to 847.61)	3.97 (2.53 to 5.70)	682.12 (422.27 to 990.37)	3.40 (2.09 to 4.95)	-0.53 (-1.19 to 0.13)	0.117
Kenya	38.09 (16.87 to 64.18)	0.39 (0.18 to 0.65)	117.63 (66.48 to 182.56)	0.41 (0.23 to 0.62)	0.15 (0.03 to 0.28)	0.018	1423.03 (597.26 to 2404.99)	12.98 (5.71 to 21.92)	4434.24 (2426.53 to 6955.23)	13.75 (7.74 to 21.37)	0.19 (0.08 to 0.31)	0.001
Kiribati	0.03 (0.01 to 0.08)	0.07 (0.01 to 0.17)	0.03 (0.00 to 0.09)	0.03 (0.00 to 0.10)	-2.43 (-2.62 to -2.25)	<0.001	1.23 (0.22 to 2.91)	2.56 (0.45 to 6.10)	1.23 (0.12 to 3.53)	1.25 (0.12 to 3.58)	-2.35 (-2.53 to -2.18)	<0.001
Kyrgyzstan	2.71 (1.54 to 4.18)	0.08 (0.05 to 0.13)	4.77 (2.66 to 7.67)	0.08 (0.04 to 0.13)	-0.17 (-0.5 to 0.16)	0.324	106.27 (61.46 to 162.78)	3.22 (1.86 to 4.93)	188.93 (107.92 to 299.96)	3.01 (1.70 to 4.81)	-0.24 (-0.55 to 0.07)	0.129
Lao People's Democratic Republic	10.02 (3.94 to 18.39)	0.40 (0.16 to 0.74)	23.80 (12.31 to 40.07)	0.43 (0.22 to 0.72)	0.19 (0.05 to 0.33)	0.007	386.71 (153.11 to 707.47)	14.45 (5.72 to 26.45)	896.77 (465.58 to 1511.54)	14.56 (7.54 to 24.52)	0.03 (-0.11 to 0.17)	0.679
Latvia	7.97 (4.63 to 11.82)	0.24 (0.14 to 0.35)	3.52 (1.96 to 5.37)	0.11 (0.06 to 0.17)	-2.46 (-3.76 to -1.14)	<0.001	287.16 (173.42 to 418.92)	8.81 (5.36 to 12.86)	109.37 (63.52 to 163.06)	3.87 (2.30 to 5.72)	-2.76 (-4.12 to -1.38)	<0.001
Lebanon	1.53 (0.61 to 2.93)	0.06 (0.03 to 0.12)	1.55 (0.68 to 2.89)	0.03 (0.01 to 0.05)	-2.73 (-2.86 to -2.6)	<0.001	56.22 (22.77 to 107.65)	2.21 (0.89 to 4.22)	54.86 (24.84 to 100.92)	0.94 (0.42 to 1.72)	-2.71 (-2.82 to -2.59)	<0.001
Lesotho	0.90 (0.37 to 1.69)	0.10 (0.04 to 0.18)	2.29 (0.96 to 4.21)	0.18 (0.08 to 0.33)	2.05 (1.76 to 2.33)	<0.001	30.83 (12.45 to 58.59)	3.17 (1.29 to 6.00)	83.95 (34.96 to 155.44)	6.17 (2.57 to 11.42)	2.23 (1.99 to 2.47)	<0.001
Liberia	0.44 (0.20 to 0.79)	0.03 (0.02 to 0.06)	1.23 (0.57 to 2.23)	0.03 (0.02 to 0.06)	0.05 (-0.23 to 0.32)	0.744	18.28 (8.30 to 32.97)	1.26 (0.58 to 2.26)	55.82 (25.47 to 101.76)	1.37 (0.63 to 2.48)	0.3 (-0.04 to 0.63)	0.08
Libya	0.02 (0.00 to 0.05)	0.00 (0.00 to 0.00)	3.03 (1.19 to 6.11)	0.04 (0.02 to 0.09)	14.54 (14.01 to 15.07)	<0.001	0.94 (0.16 to 2.59)	0.03 (0.00 to 0.08)	124.72 (49.53 to 250.18)	1.61 (0.63 to 3.24)	13.93 (13.44 to 14.43)	<0.001

Lithuania	9.76（5.64 to 14.19）	0.22（0.13 to 0.33）	4.42（2.59 to 6.66）	0.10（0.06 to 0.14）	-2.7 (-3.67 to -1.71)	<0.001	354.35（213.96 to 507.17）	8.36（5.09 to 11.96）	139.31（85.74 to 205.06）	3.35（2.10 to 4.89）	-2.94 (-3.99 to -1.87)	<0.001
Luxembourg	1.45（1.01 to 1.89）	0.29（0.20 to 0.37）	1.12（0.79 to 1.50）	0.11（0.08 to 0.15）	-3.12 (-3.43 to -2.8)	<0.001	47.10（33.67 to 61.00）	9.74（6.98 to 12.59）	32.89（23.48 to 44.23）	3.46（2.47 to 4.66）	-3.31 (-3.61 to -3)	<0.001
Madagascar	9.96（2.48 to 19.46）	0.16（0.04 to 0.32）	15.15（4.79 to 32.49）	0.09（0.03 to 0.20）	-1.8 (-2 to -1.59)	<0.001	377.95（86.78 to 737.41）	5.68（1.38 to 11.10）	618.72（191.23 to 1339.15）	3.38（1.07 to 7.24）	-1.68 (-1.85 to -1.5)	<0.001
Malawi	1.77（0.57 to 3.29）	0.04（0.01 to 0.07）	5.17（2.34 to 9.69）	0.05（0.02 to 0.10）	1.08 (0.92 to 1.24)	<0.001	68.55（21.06 to 128.52）	1.30（0.42 to 2.42）	211.59（94.14 to 399.95）	1.88（0.85 to 3.51）	1.2 (1.06 to 1.34)	<0.001
Malaysia	84.60（39.74 to 151.03）	0.74（0.35 to 1.31）	104.05（46.04 to 196.81）	0.33（0.14 to 0.62）	-2.63 (-3.06 to -2.2)	<0.001	3355.21（1574.22 to 6028.68）	26.65（12.56 to 47.57）	4007.23（1783.07 to 7577.60）	12.18（5.41 to 23.02）	-2.55 (-3.06 to -2.04)	<0.001
Maldives	0.01（0.00 to 0.02）	0.01（0.00 to 0.02）	0.03（0.01 to 0.07）	0.01（0.00 to 0.01）	0.16 (-0.56 to 0.89)	0.658	0.23（0.00 to 0.77）	0.18（0.00 to 0.63）	1.17（0.31 to 2.80）	0.21（0.05 to 0.51）	0.34 (-0.39 to 1.07)	0.364
Mali	0.51（0.20 to 0.94）	0.01（0.00 to 0.02）	1.16（0.51 to 2.22）	0.01（0.00 to 0.02）	-0.14 (-0.3 to 0.03)	0.102	17.93（6.90 to 33.33）	0.36（0.14 to 0.67）	43.77（19.13 to 85.44）	0.36（0.16 to 0.69）	-0.02 (-0.17 to 0.14)	0.838
Malta	1.52（0.89 to 2.24）	0.36（0.21 to 0.53）	1.68（1.04 to 2.54）	0.22（0.14 to 0.34）	-1.52 (-1.96 to -1.07)	<0.001	49.30（29.99 to 72.06）	11.78（7.19 to 17.20）	49.87（31.36 to 75.12）	7.58（4.79 to 11.42）	-1.41 (-1.67 to -1.14)	<0.001
Marshall Islands	0.02（0.01 to 0.04）	0.10（0.03 to 0.21）	0.05（0.02 to 0.10）	0.11（0.04 to 0.22）	0.27 (0.18 to 0.36)	<0.001	0.77（0.25 to 1.60）	3.56（1.14 to 7.41）	1.85（0.67 to 3.79）	3.77（1.36 to 7.69）	0.19 (0.14 to 0.24)	<0.001
Mauritius	1.35（0.75 to 1.89）	0.17（0.09 to 0.23）	2.60（1.62 to 3.73）	0.14（0.09 to 0.20）	-0.4 (-0.92 to 0.11)	0.127	47.75（26.44 to 66.95）	5.48（3.04 to 7.67）	85.37（53.63 to 121.74）	4.81（3.01 to 6.86）	-0.3 (-0.9 to 0.3)	0.327
Mexico	18.53（13.04 to 24.62）	0.04（0.03 to 0.05）	41.11（27.85 to 56.01）	0.03（0.02 to 0.04）	-0.63 (-1.08 to -0.18)	0.006	680.14（485.28 to 893.05）	1.23（0.87 to 1.62）	1397.95（964.01 to 1881.95）	1.03（0.71 to 1.39）	-0.54 (-0.98 to -0.11)	0.015
Micronesia (Federated States of)	0.13（0.05 to 0.24）	0.24（0.09 to 0.44）	0.09（0.03 to 0.18）	0.10（0.04 to 0.20）	-2.67 (-2.75 to -2.58)	<0.001	4.64（1.80 to 8.64）	8.15（3.17 to 15.12）	3.45（1.22 to 6.71）	3.61（1.27 to 7.04）	-2.62 (-2.69 to -2.55)	<0.001
Monaco	0.03（0.00 to 0.07）	0.06（0.00 to 0.12）	0.04（0.00 to 0.08）	0.05（0.00 to 0.11）	-0.37 (-0.46 to -0.28)	<0.001	0.88（0.03 to 1.90）	1.93（0.08 to 4.09）	1.04（0.05 to 2.20）	1.73（0.11 to 3.68）	-0.35 (-0.46 to -0.25)	<0.001
Mongolia	0.69（0.29 to 1.29）	0.05（0.02 to 0.10）	3.17（1.74 to 5.17）	0.11（0.06 to 0.18）	2.19 (1.99 to 2.39)	<0.001	27.63（12.22 to 51.55）	2.00（0.87 to 3.73）	123.89（69.40 to 201.63）	3.90（2.14 to 6.37）	2.18 (1.94 to 2.42)	<0.001
Montenegro	0.23（0.14 to 0.32）	0.03（0.02 to 0.05）	0.32（0.20 to 0.46）	0.03（0.02 to 0.05）	0.06 (-0.21 to 0.33)	0.681	7.98（5.10 to 11.27）	1.20（0.77 to 1.70）	10.04（6.49 to 14.43）	1.15（0.74 to 1.67）	-0.09 (-0.37 to 0.19)	0.53
Morocco	5.42（2.29 to 10.47）	0.03（0.01 to 0.06）	6.89（2.78 to 13.80）	0.02（0.01 to 0.03）	-1.64 (-1.79 to -1.49)	<0.001	257.51（109.20 to 499.19）	1.30（0.55 to 2.50）	292.18（118.49 to 586.17）	0.73（0.30 to 1.47）	-1.84 (-1.97 to -1.71)	<0.001
Mozambique	0.21（0.01 to 0.49）	0.00（0.00 to 0.01）	1.73（0.57 to 3.39）	0.01（0.00 to 0.02）	4.82 (4.64 to 4.99)	<0.001	7.54（0.18 to 17.99）	0.10（0.00 to 0.23）	65.83（20.95 to 130.51）	0.41（0.14 to 0.81）	4.81 (4.61 to 5)	<0.001
Myanmar	10.65	0.04	102.20	0.19	5.35 (5.2 to	<0.001	426.42	1.42	3690.42	6.52	5.08 (4.94 to	<0.001

	（2.38 to 23.71）	（0.01 to 0.08）	（51.62 to 180.34）	（0.09 to 0.33）	5.49)		（94.98 to 939.46）	（0.32 to 3.16）	（1856.99 to 6490.48）	（3.28 to 11.47）	5.21)	
Namibia	0.90（0.34 to 1.66）	0.11（0.04 to 0.21）	2.68（1.34 to 4.48）	0.16（0.08 to 0.26）	1.06 (0.81 to 1.31)	<0.001	35.02（13.05 to 64.78）	4.08（1.51 to 7.51）	103.81（50.82 to 176.31）	5.52（2.75 to 9.26）	1.01 (0.75 to 1.28)	<0.001
Nauru	0.01（0.00 to 0.03）	0.26（0.08 to 0.56）	0.02（0.01 to 0.03）	0.23（0.09 to 0.48）	-0.42 (-0.49 to -0.36)	<0.001	0.55（0.17 to 1.19）	8.83（2.72 to 18.87）	0.61（0.22 to 1.28）	7.86（2.88 to 16.40）	-0.37 (-0.44 to -0.31)	<0.001
Nepal	1.53（0.01 to 4.75）	0.01（0.00 to 0.04）	33.78（11.62 to 66.32）	0.13（0.04 to 0.25）	7.98 (7.8 to 8.16)	<0.001	65.56（0.72 to 199.46）	0.48（0.00 to 1.48）	1263.88（440.76 to 2467.38）	4.62（1.60 to 9.03）	7.61 (7.44 to 7.77)	<0.001
Netherlands	28.01（18.49 to 37.63）	0.15（0.10 to 0.20）	26.49（17.51 to 37.08）	0.08（0.06 to 0.12）	-1.89 (-2.07 to -1.71)	<0.001	876.53（595.00 to 1169.33）	4.90（3.35 to 6.53）	718.40（479.89 to 1004.18）	2.59（1.72 to 3.65）	-2.07 (-2.26 to -1.88)	<0.001
New Zealand	5.21（3.06 to 7.63）	0.14（0.08 to 0.20）	4.70（3.16 to 6.59）	0.06（0.04 to 0.09）	-2.55 (-3.08 to -2.02)	<0.001	179.72（111.72 to 257.67）	4.93（3.09 to 7.04）	149.29（102.20 to 209.81）	2.20（1.51 to 3.10）	-2.6 (-3.14 to -2.06)	<0.001
Nicaragua	0.58（0.31 to 0.96）	0.03（0.02 to 0.05）	1.87（1.00 to 3.15）	0.03（0.02 to 0.06）	0.3 (0 to 0.6)	0.05	22.24（12.17 to 36.79）	1.05（0.58 to 1.74）	66.54（36.26 to 111.84）	1.15（0.62 to 1.93）	0.3 (-0.02 to 0.62)	0.07
Niger	0.04（0.00 to 0.11）	0.00（0.00 to 0.00）	0.30（0.05 to 0.82）	0.00（0.00 to 0.01）	3.28 (3.06 to 3.51)	<0.001	1.78（0.06 to 5.15）	0.04（0.00 to 0.11）	13.56（2.46 to 37.33）	0.10（0.02 to 0.28）	3.27 (3.05 to 3.49)	<0.001
Nigeria	102.20（55.11 to 156.72）	0.20（0.11 to 0.31）	232.78（133.28 to 365.89）	0.20（0.12 to 0.31）	-0.08 (-0.18 to 0.03)	0.155	3748.02（1989.02 to 5779.04）	6.86（3.68 to 10.54）	8966.86（5057.26 to 14265.29）	6.71（3.84 to 10.55）	-0.12 (-0.23 to 0)	0.046
Niue	0.00（0.00 to 0.01）	0.17（0.03 to 0.33）	0.00（0.00 to 0.01）	0.16（0.06 to 0.30）	-0.15 (-0.32 to 0.02)	0.085	0.12（0.02 to 0.23）	5.77（1.10 to 11.37）	0.12（0.05 to 0.22）	5.53（2.18 to 10.28）	-0.17 (-0.36 to 0.02)	0.073
North Macedonia	2.06（1.33 to 2.92）	0.10（0.07 to 0.15）	3.12（1.95 to 4.51）	0.09（0.06 to 0.14）	-0.24 (-0.6 to 0.11)	0.182	70.61（46.54 to 100.40）	3.42（2.24 to 4.87）	97.73（61.32 to 141.96）	3.04（1.89 to 4.44）	-0.35 (-0.7 to 0)	0.052
Northern Mariana Islands	0.04（0.00 to 0.10）	0.16（0.00 to 0.39）	0.13（0.02 to 0.29）	0.22（0.03 to 0.46）	1.12 (0.67 to 1.57)	<0.001	1.65（0.03 to 4.11）	5.26（0.07 to 13.21）	4.63（0.61 to 9.74）	7.12（0.95 to 15.04）	0.97 (0.55 to 1.4)	<0.001
Norway	2.85（1.61 to 4.01）	0.05（0.03 to 0.07）	2.19（1.50 to 2.98）	0.02（0.02 to 0.03）	-2.29 (-2.84 to -1.74)	<0.001	88.05（53.31 to 121.81）	1.71（1.07 to 2.34）	61.64（43.22 to 83.69）	0.79（0.56 to 1.08）	-2.5 (-3.07 to -1.94)	<0.001
Oman	0.07（0.02 to 0.15）	0.01（0.00 to 0.01）	0.17（0.06 to 0.36）	0.00（0.00 to 0.01）	-1.26 (-1.54 to -0.99)	<0.001	3.12（1.08 to 6.54）	0.25（0.08 to 0.53）	7.46（2.69 to 16.01）	0.17（0.06 to 0.36）	-1.32 (-1.66 to -0.98)	<0.001
Pakistan	18.22（2.07 to 37.60）	0.03（0.00 to 0.06）	78.38（25.82 to 155.88）	0.05（0.01 to 0.09）	1.75 (1.63 to 1.86)	<0.001	738.14（84.04 to 1519.14）	1.03（0.12 to 2.13）	3290.65（1118.54 to 6508.24）	1.80（0.59 to 3.57）	1.78 (1.67 to 1.9)	<0.001
Palau	0.00（0.00 to 0.00）	0.00（0.00 to 0.01）	0.00（0.00 to 0.00）	0.00（0.00 to 0.01）	-0.04 (-0.31 to 0.23)	0.773	0.01（0.00 to 0.03）	0.07（0.00 to 0.20）	0.02（0.00 to 0.04）	0.07（0.01 to 0.19）	0.07 (-0.16 to 0.3)	0.563
Palestine	0.14（0.06 to 0.28）	0.01（0.01 to 0.03）	0.28（0.13 to 0.53）	0.01（0.00 to 0.02）	-1.66 (-1.88 to -1.44)	<0.001	5.82（2.43 to 11.27）	0.49（0.20 to 0.94）	11.59（5.33 to 21.51）	0.30（0.14 to 0.56）	-1.6 (-1.93 to -1.27)	<0.001
Panama	1.02（0.68 to 1.41）	0.06（0.04 to 0.09）	2.63（1.61 to 3.83）	0.06（0.04 to 0.09）	-0.2 (-0.52 to 0.13)	0.239	34.58（23.57 to 47.58）	1.97（1.34 to 2.72）	83.04（52.22 to 119.90）	1.88（1.18 to 2.71）	-0.14 (-0.87 to 0.59)	0.709

Papua New Guinea	1.62（0.60 to 3.20）	0.07（0.03 to 0.14）	3.01（1.00 to 6.45）	0.04（0.01 to 0.10）	-1.43 (-1.64 to -1.22)	<0.001	61.88（22.68 to 122.16）	2.44（0.90 to 4.83）	114.91（38.55 to 247.09）	1.54（0.51 to 3.31）	-1.5 (-1.72 to -1.27)	<0.001
Paraguay	0.90（0.55 to 1.35）	0.04（0.02 to 0.05）	3.38（1.83 to 5.70）	0.05（0.03 to 0.09）	1.31 (0.92 to 1.7)	<0.001	34.96（21.37 to 52.62）	1.27（0.77 to 1.91）	125.84（68.44 to 212.58）	1.89（1.03 to 3.19）	1.31 (1.03 to 1.58)	<0.001
Peru	3.77（1.87 to 6.25）	0.03（0.01 to 0.05）	8.29（4.39 to 14.12）	0.02（0.01 to 0.04）	-0.48 (-1.36 to 0.41)	0.287	136.00（67.31 to 224.11）	0.91（0.45 to 1.50）	264.02（140.84 to 447.12）	0.74（0.39 to 1.25）	-0.63 (-1.48 to 0.24)	0.154
Philippines	147.84（94.58 to 202.43）	0.40（0.25 to 0.55）	407.43（286.67 to 551.94）	0.43（0.30 to 0.58）	0.22 (0.01 to 0.44)	0.043	5917.02（3789.25 to 8039.99）	14.15（9.07 to 19.34）	15069.38（10644.61 to 20348.43）	14.80（10.44 to 20.01）	0.15 (-0.13 to 0.43)	0.293
Poland	50.83（34.99 to 65.24）	0.12（0.08 to 0.15）	70.92（51.26 to 89.95）	0.11（0.08 to 0.14）	-0.2 (-0.74 to 0.33)	0.458	1768.23（1259.77 to 2243.35）	4.19（3.00 to 5.31）	2191.41（1622.14 to 2754.18）	3.86（2.87 to 4.84）	-0.36 (-0.93 to 0.22)	0.226
Portugal	32.28（21.23 to 43.46）	0.25（0.17 to 0.34）	27.77（18.43 to 39.87）	0.14（0.10 to 0.20）	-1.9 (-2.41 to -1.38)	<0.001	1057.14（711.47 to 1421.06）	8.64（5.85 to 11.63）	811.95（541.35 to 1159.25）	4.72（3.15 to 6.74）	-2.05 (-2.56 to -1.54)	<0.001
Puerto Rico	3.42（2.04 to 5.10）	0.10（0.06 to 0.14）	4.25（2.48 to 6.70）	0.08（0.04 to 0.12）	-0.75 (-1.18 to -0.32)	0.001	116.68（72.06 to 170.91）	3.27（2.02 to 4.79）	125.33（74.59 to 195.70）	2.61（1.57 to 4.06）	-0.72 (-1.13 to -0.31)	0.001
Qatar	0.03（0.01 to 0.05）	0.01（0.00 to 0.02）	0.14（0.05 to 0.28）	0.01（0.00 to 0.01）	-1.93 (-2.66 to -1.19)	<0.001	1.24（0.50 to 2.46）	0.37（0.15 to 0.74）	6.46（2.60 to 12.88）	0.21（0.08 to 0.43）	-1.76 (-2.61 to -0.9)	<0.001
Republic of Korea	58.80（35.51 to 89.32）	0.17（0.10 to 0.26）	70.41（44.25 to 104.69）	0.08（0.05 to 0.12）	-2.43 (-2.61 to -2.26)	<0.001	2172.31（1307.46 to 3308.31）	5.58（3.36 to 8.48）	2085.60（1315.33 to 3092.29）	2.48（1.55 to 3.69）	-2.6 (-2.79 to -2.41)	<0.001
Republic of Moldova	11.92（7.65 to 15.81）	0.26（0.17 to 0.35）	13.61（8.59 to 18.56）	0.24（0.16 to 0.33）	-0.07 (-0.65 to 0.51)	0.817	417.06（279.80 to 545.46）	9.04（6.06 to 11.84）	440.16（289.96 to 588.36）	8.23（5.50 to 10.97）	-0.36 (-1.01 to 0.29)	0.277
Romania	33.80（22.12 to 46.42）	0.12（0.08 to 0.17）	76.42（48.82 to 109.83）	0.25（0.16 to 0.36）	2.33 (1.84 to 2.82)	<0.001	1197.44（800.96 to 1633.19）	4.44（2.96 to 6.08）	2452.99（1589.06 to 3516.40）	8.65（5.61 to 12.41）	2.26 (1.79 to 2.73)	<0.001
Russian Federation	186.91（122.01 to 241.23）	0.10（0.07 to 0.14）	188.75（120.84 to 252.54）	0.09（0.06 to 0.12）	-0.36 (-1.46 to 0.76)	0.529	6817.27（4632.76 to 8695.22）	3.91（2.68 to 4.99）	6452.39（4324.22 to 8463.15）	3.19（2.19 to 4.15）	-0.47 (-1.55 to 0.63)	0.401
Rwanda	23.18（9.98 to 38.92）	0.65（0.29 to 1.10）	29.47（14.09 to 52.87）	0.36（0.18 to 0.64）	-1.92 (-2.09 to -1.74)	<0.001	915.10（372.94 to 1545.36）	23.26（10.01 to 39.13）	1160.89（540.94 to 2109.62）	12.67（6.04 to 22.75）	-1.91 (-2.09 to -1.73)	<0.001
Saint Kitts and Nevis	0.04（0.00 to 0.06）	0.11（0.00 to 0.16）	0.06（0.00 to 0.15）	0.08（0.00 to 0.20）	-0.76 (-1.3 to -0.23)	0.005	1.15（0.02 to 1.76）	3.56（0.06 to 5.40）	2.05（0.02 to 4.89）	2.60（0.03 to 6.23）	-0.94 (-1.41 to -0.47)	<0.001
Saint Lucia	0.17（0.11 to 0.22）	0.19（0.13 to 0.25）	0.47（0.31 to 0.67）	0.20（0.13 to 0.28）	0.17 (-0.14 to 0.48)	0.28	5.82（4.11 to 7.70）	6.12（4.32 to 8.13）	15.08（10.03 to 21.44）	6.42（4.28 to 9.11）	0.17 (-0.08 to 0.42)	0.195
Saint Vincent and the Grenadines	0.06（0.03 to 0.09）	0.08（0.05 to 0.12）	0.23（0.16 to 0.32）	0.17（0.11 to 0.23）	2.35 (2.09 to 2.61)	<0.001	2.13（1.31 to 3.04）	2.75（1.70 to 3.94）	7.56（5.19 to 10.41）	5.43（3.72 to 7.47）	2.17 (1.96 to 2.39)	<0.001
Samoa	0.23（0.09 to 0.43）	0.23（0.09 to 0.43）	0.25（0.09 to 0.47）	0.15（0.06 to 0.28）	-1.43 (-1.52 to -1.34)	<0.001	8.83（3.42 to 16.58）	8.38（3.23 to 15.69）	9.55（3.71 to 18.22）	5.56（2.16 to 10.62）	-1.32 (-1.4 to -1.24)	<0.001
San Marino	0.07	0.23	0.06	0.10	-2.67 (-3.05 to -	<0.001	2.19	7.34	1.80	3.39	-2.64 (-2.92 to -	<0.001

	(0.00 to 0.13)	(0.00 to 0.41)	(0.00 to 0.13)	(0.00 to 0.21)	2.29)		(0.02 to 3.94)	(0.07 to 13.16)	(0.02 to 3.61)	(0.05 to 6.79)	2.36)	
Sao Tome and Principe	0.00 (0.00 to 0.00)	0.00 (0.00 to 0.01)	0.01 (0.00 to 0.01)	0.00 (0.00 to 0.01)	0.62 (0.15 to 1.08)	0.009	0.08 (0.03 to 0.15)	0.12 (0.05 to 0.21)	0.25 (0.11 to 0.47)	0.14 (0.06 to 0.26)	0.51 (0.07 to 0.95)	0.024
Saudi Arabia	1.89 (0.45 to 4.10)	0.02 (0.00 to 0.05)	2.65 (0.35 to 6.55)	0.01 (0.00 to 0.02)	-3.55 (-3.87 to -3.23)	<0.001	81.98 (20.51 to 177.14)	0.81 (0.19 to 1.76)	118.48 (16.85 to 290.21)	0.27 (0.04 to 0.69)	-3.48 (-3.8 to -3.15)	<0.001
Senegal	0.25 (0.03 to 0.59)	0.01 (0.00 to 0.01)	0.48 (0.11 to 1.22)	0.00 (0.00 to 0.01)	-0.97 (-1.21 to -0.73)	<0.001	10.72 (1.45 to 25.07)	0.24 (0.03 to 0.57)	21.67 (4.89 to 54.50)	0.19 (0.04 to 0.48)	-0.75 (-1.02 to -0.48)	<0.001
Serbia	14.65 (8.04 to 24.96)	0.12 (0.07 to 0.21)	14.80 (8.39 to 23.46)	0.10 (0.06 to 0.16)	-0.62 (-0.9 to -0.34)	<0.001	499.02 (278.59 to 843.74)	4.22 (2.34 to 7.13)	447.67 (258.13 to 708.28)	3.38 (1.95 to 5.37)	-0.68 (-1.1 to -0.25)	0.002
Seychelles	0.19 (0.09 to 0.32)	0.34 (0.16 to 0.57)	0.41 (0.22 to 0.70)	0.32 (0.17 to 0.55)	-0.13 (-0.4 to 0.15)	0.371	6.82 (3.31 to 11.47)	12.03 (5.89 to 20.19)	14.66 (7.77 to 24.94)	11.19 (5.90 to 19.10)	-0.16 (-0.33 to 0.02)	0.076
Sierra Leone	0.65 (0.30 to 1.14)	0.03 (0.01 to 0.05)	1.48 (0.66 to 2.70)	0.03 (0.01 to 0.05)	-0.04 (-0.26 to 0.17)	0.681	26.21 (11.73 to 46.07)	1.05 (0.47 to 1.83)	64.11 (28.49 to 117.95)	1.10 (0.49 to 2.00)	0.14 (-0.1 to 0.37)	0.253
Singapore	19.31 (11.03 to 29.84)	0.68 (0.39 to 1.06)	17.97 (9.76 to 29.48)	0.21 (0.11 to 0.35)	-3.65 (-4.08 to -3.22)	<0.001	782.32 (448.34 to 1206.70)	25.68 (14.69 to 39.64)	590.70 (321.61 to 961.07)	7.17 (3.86 to 11.70)	-3.99 (-4.43 to -3.54)	<0.001
Slovakia	17.06 (10.10 to 25.32)	0.30 (0.18 to 0.44)	14.80 (8.50 to 24.08)	0.17 (0.10 to 0.28)	-1.76 (-2.16 to -1.36)	<0.001	601.36 (360.43 to 892.05)	10.72 (6.44 to 15.89)	467.82 (269.76 to 763.71)	5.80 (3.34 to 9.52)	-1.95 (-2.4 to -1.49)	<0.001
Slovenia	4.03 (2.53 to 5.71)	0.17 (0.10 to 0.24)	1.29 (0.58 to 2.28)	0.03 (0.02 to 0.06)	-5.2 (-5.78 to -4.62)	<0.001	136.17 (87.51 to 191.67)	5.72 (3.68 to 8.06)	36.98 (17.37 to 64.08)	1.10 (0.52 to 1.90)	-5.44 (-6.06 to -4.81)	<0.001
Solomon Islands	0.04 (0.01 to 0.11)	0.03 (0.00 to 0.06)	0.21 (0.06 to 0.43)	0.04 (0.01 to 0.09)	1.87 (1.49 to 2.26)	<0.001	1.76 (0.21 to 4.27)	0.92 (0.11 to 2.25)	8.17 (2.26 to 17.00)	1.63 (0.44 to 3.39)	1.87 (1.49 to 2.24)	<0.001
South Africa	37.51 (22.22 to 52.22)	0.16 (0.10 to 0.23)	66.25 (42.27 to 88.51)	0.13 (0.08 to 0.17)	-0.71 (-1.07 to -0.34)	<0.001	1365.84 (815.01 to 1882.10)	5.37 (3.17 to 7.44)	2264.99 (1428.20 to 3036.23)	4.20 (2.66 to 5.62)	-0.86 (-1.23 to -0.48)	<0.001
South Sudan	0.63 (0.01 to 1.73)	0.02 (0.00 to 0.06)	0.77 (0.08 to 2.32)	0.01 (0.00 to 0.04)	-1.34 (-1.5 to -1.18)	<0.001	24.59 (0.20 to 67.29)	0.74 (0.01 to 2.06)	32.29 (2.99 to 96.91)	0.51 (0.05 to 1.55)	-1.2 (-1.39 to -1.02)	<0.001
Spain	126.75 (85.03 to 169.81)	0.26 (0.18 to 0.35)	90.30 (59.60 to 128.46)	0.11 (0.07 to 0.16)	-2.81 (-3.12 to -2.51)	<0.001	4210.00 (2906.69 to 5627.32)	9.12 (6.36 to 12.18)	2645.54 (1762.54 to 3750.94)	3.62 (2.41 to 5.14)	-3.07 (-3.43 to -2.71)	<0.001
Sri Lanka	14.24 (6.53 to 25.19)	0.12 (0.05 to 0.21)	27.30 (12.59 to 50.17)	0.10 (0.05 to 0.18)	-0.43 (-0.62 to -0.24)	<0.001	516.78 (235.96 to 919.39)	3.80 (1.74 to 6.75)	892.52 (414.06 to 1644.58)	3.32 (1.54 to 6.12)	-0.36 (-1.01 to 0.3)	0.282
Sudan	4.46 (1.77 to 9.00)	0.04 (0.02 to 0.08)	0.00 (0.00 to 0.02)	0.00 (0.00 to 0.00)	-25.12 (-26.34 to -23.88)	<0.001	173.08 (69.18 to 347.65)	1.43 (0.56 to 2.87)	0.13 (0.00 to 1.03)	0.00 (0.00 to 0.00)	-24.08 (-25.19 to -22.94)	<0.001
Suriname	0.35 (0.19 to 0.54)	0.12 (0.07 to 0.19)	0.84 (0.43 to 1.47)	0.13 (0.06 to 0.22)	0.23 (-0.34 to 0.8)	0.435	12.80 (7.28 to 19.81)	4.10 (2.33 to 6.36)	28.99 (15.19 to 50.52)	4.37 (2.29 to 7.60)	0.25 (-0.35 to 0.87)	0.411
Sweden	6.41 (3.85 to 9.20)	0.05 (0.03 to 0.07)	5.09 (3.28 to 7.41)	0.03 (0.02 to 0.04)	-1.85 (-2.9 to -0.78)	0.001	189.07 (120.46 to 268.17)	1.67 (1.09 to 2.35)	134.56 (87.90 to 194.11)	0.88 (0.58 to 1.27)	-2 (-3.05 to -0.94)	<0.001

Switzerland	22.11 (14.45 to 30.05)	0.24 (0.16 to 0.33)	9.60 (6.03 to 14.06)	0.06 (0.04 to 0.09)	-4.52 (-5.02 to -4.01)	<0.001	696.25 (469.50 to 938.80)	8.00 (5.44 to 10.78)	261.20 (166.15 to 381.24)	1.84 (1.18 to 2.69)	-4.83 (-5.38 to -4.27)	<0.001
Syrian Arab Republic	0.48 (0.23 to 0.84)	0.01 (0.00 to 0.01)	0.35 (0.14 to 0.67)	0.00 (0.00 to 0.00)	-3.66 (-4.03 to -3.29)	<0.001	19.10 (9.40 to 33.12)	0.26 (0.13 to 0.46)	12.62 (5.15 to 24.00)	0.08 (0.03 to 0.15)	-3.77 (-4.15 to -3.38)	<0.001
Taiwan (Province of China)	256.43 (185.83 to 340.60)	1.41 (1.02 to 1.88)	184.03 (115.08 to 268.35)	0.47 (0.30 to 0.69)	-3.54 (-3.78 to -3.3)	<0.001	9887.72 (7194.71 to 13096.06)	52.31 (38.01 to 69.26)	6295.87 (3983.66 to 9131.45)	17.08 (10.81 to 24.84)	-3.61 (-3.85 to -3.37)	<0.001
Tajikistan	1.95 (0.94 to 3.57)	0.06 (0.03 to 0.11)	2.93 (1.44 to 5.16)	0.03 (0.02 to 0.06)	-1.68 (-1.93 to -1.43)	<0.001	82.51 (39.56 to 151.71)	2.30 (1.11 to 4.23)	129.97 (64.34 to 228.10)	1.43 (0.70 to 2.52)	-1.53 (-1.81 to -1.25)	<0.001
Thailand	157.99 (85.33 to 255.87)	0.37 (0.20 to 0.59)	317.24 (176.12 to 519.14)	0.31 (0.17 to 0.51)	-0.54 (-0.72 to -0.36)	<0.001	6091.74 (3294.71 to 9939.08)	12.89 (6.99 to 20.92)	10871.53 (6080.77 to 17786.55)	11.31 (6.33 to 18.53)	-0.42 (-0.59 to -0.24)	<0.001
Timor-Leste	0.47 (0.17 to 1.03)	0.12 (0.04 to 0.26)	1.78 (0.81 to 3.30)	0.19 (0.09 to 0.36)	1.51 (1.31 to 1.7)	<0.001	18.81 (6.58 to 41.40)	4.15 (1.46 to 9.07)	61.53 (27.81 to 114.45)	6.36 (2.89 to 11.79)	1.41 (1.08 to 1.73)	<0.001
Togo	0.37 (0.14 to 0.68)	0.02 (0.01 to 0.04)	1.50 (0.66 to 2.81)	0.03 (0.01 to 0.05)	0.65 (0.37 to 0.94)	<0.001	15.90 (6.05 to 29.24)	0.82 (0.32 to 1.48)	64.70 (28.14 to 121.67)	1.03 (0.45 to 1.93)	0.8 (0.5 to 1.11)	<0.001
Tokelau	0.00 (0.00 to 0.00)	0.11 (0.03 to 0.25)	0.00 (0.00 to 0.00)	0.11 (0.04 to 0.23)	-0.01 (-0.09 to 0.07)	0.821	0.05 (0.01 to 0.11)	3.81 (1.05 to 8.24)	0.05 (0.02 to 0.11)	3.82 (1.50 to 7.72)	0.04 (-0.09 to 0.16)	0.566
Tonga	0.03 (0.01 to 0.06)	0.05 (0.01 to 0.10)	0.03 (0.01 to 0.06)	0.03 (0.01 to 0.07)	-1.33 (-1.45 to -1.21)	<0.001	0.97 (0.27 to 2.08)	1.52 (0.43 to 3.27)	0.88 (0.21 to 2.00)	1.02 (0.24 to 2.31)	-1.28 (-1.37 to -1.19)	<0.001
Trinidad and Tobago	0.57 (0.36 to 0.80)	0.07 (0.04 to 0.09)	1.62 (0.99 to 2.44)	0.09 (0.05 to 0.13)	1.15 (0.9 to 1.4)	<0.001	19.77 (12.94 to 27.18)	2.12 (1.38 to 2.92)	52.50 (32.53 to 79.04)	2.91 (1.81 to 4.38)	1.28 (1.03 to 1.53)	<0.001
Tunisia	7.89 (3.42 to 14.87)	0.13 (0.06 to 0.25)	17.75 (7.69 to 34.36)	0.13 (0.06 to 0.25)	-0.15 (-0.26 to -0.04)	0.007	316.14 (140.16 to 594.39)	5.05 (2.22 to 9.50)	687.91 (303.21 to 1316.24)	4.97 (2.19 to 9.50)	-0.06 (-0.15 to 0.04)	0.257
Turkey	30.95 (13.94 to 56.53)	0.07 (0.03 to 0.13)	38.33 (17.56 to 67.83)	0.04 (0.02 to 0.07)	-1.99 (-2.19 to -1.8)	<0.001	1274.75 (585.50 to 2314.61)	2.77 (1.26 to 5.03)	1365.96 (638.11 to 2393.10)	1.40 (0.66 to 2.46)	-2.2 (-2.38 to -2.02)	<0.001
Turkmenistan	1.14 (0.54 to 1.81)	0.05 (0.02 to 0.08)	4.47 (2.58 to 6.73)	0.09 (0.05 to 0.14)	2.1 (1.14 to 3.07)	<0.001	46.89 (23.35 to 73.63)	1.82 (0.88 to 2.87)	177.37 (105.59 to 265.41)	3.51 (2.07 to 5.27)	2.12 (1.13 to 3.12)	<0.001
Tuvalu	0.01 (0.00 to 0.01)	0.09 (0.02 to 0.19)	0.01 (0.00 to 0.02)	0.09 (0.03 to 0.17)	-0.1 (-0.25 to 0.05)	0.197	0.24 (0.07 to 0.51)	3.03 (0.84 to 6.52)	0.34 (0.12 to 0.67)	2.97 (1.07 to 5.81)	-0.08 (-0.23 to 0.07)	0.282
Uganda	51.27 (18.74 to 88.10)	0.66 (0.25 to 1.12)	142.35 (71.48 to 242.12)	0.71 (0.36 to 1.20)	0.22 (-0.01 to 0.44)	0.061	1968.17 (678.30 to 3411.11)	23.00 (8.31 to 39.46)	5825.16 (2876.55 to 9978.29)	25.20 (12.70 to 42.77)	0.26 (0.03 to 0.49)	0.024
Ukraine	62.69 (34.11 to 102.56)	0.09 (0.05 to 0.15)	85.07 (42.05 to 145.18)	0.13 (0.06 to 0.22)	1.08 (0.33 to 1.84)	0.005	2222.92 (1248.35 to 3619.64)	3.37 (1.91 to 5.49)	2945.21 (1498.50 to 4946.54)	4.70 (2.43 to 7.84)	1.13 (0.35 to 1.91)	0.004
United Arab Emirates	0.89 (0.35 to 1.83)	0.09 (0.03 to 0.20)	3.00 (1.25 to 5.66)	0.04 (0.01 to 0.07)	-3.23 (-3.57 to -2.88)	<0.001	40.96 (16.45 to 83.07)	3.24 (1.22 to 6.76)	129.02 (54.36 to 242.86)	1.15 (0.46 to 2.21)	-3.32 (-3.56 to -3.08)	<0.001
United Kingdom	82.81	0.11	83.98	0.08	-1.06 (-1.32 to -	<0.001	2548.29	3.55	2433.41	2.53	-1.11 (-1.42 to -	<0.001

	（55.29 to 106.92）	（0.07 to 0.14）	（61.12 to 106.25）	（0.06 to 0.10）	0.8)		（1794.37 to 3243.07）	（2.55 to 4.49）	（1801.67 to 3046.97）	（1.88 to 3.17）	0.81)	
United Republic of Tanzania	41.79（17.62 to 73.47）	0.33（0.14 to 0.57）	89.51（42.76 to 158.29）	0.29（0.14 to 0.50）	-0.46 (-0.54 to -0.37)	<0.001	1526.31（609.90 to 2725.69）	11.08（4.59 to 19.57）	3353.04（1548.60 to 6056.67）	9.57（4.55 to 16.98）	-0.47 (-0.55 to -0.4)	<0.001
United States Virgin Islands	0.10（0.00 to 0.22）	0.09（0.06 to 0.12）	0.13（0.01 to 0.26）	0.06（0.05 to 0.08）	-1.2 (-1.39 to -1.01)	<0.001	3.41（0.02 to 7.65）	3.26（2.21 to 4.34）	3.75（0.23 to 7.77）	2.16（1.59 to 2.73）	-0.07 (-0.28 to 0.15)	<0.001
United States of America	256.79（164.15 to 355.30）	0.10（0.00 to 0.24）	319.78（229.31 to 409.70）	0.09（0.01 to 0.19）	-0.48 (-0.71 to -0.24)	<0.001	9004.32（6068.13 to 12036.81）	3.38（0.02 to 7.65）	9964.80（7306.26 to 12661.68）	3.26（0.24 to 6.84）	-1.35 (-1.57 to -1.13)	0.529
Uruguay	6.76（4.41 to 9.72）	0.18（0.12 to 0.26）	4.78（3.07 to 7.11）	0.10（0.06 to 0.15）	-2.04 (-2.6 to -1.47)	<0.001	209.71（139.55 to 299.18）	5.95（3.96 to 8.49）	140.65（91.11 to 208.28）	3.21（2.08 to 4.74）	-2.01 (-2.47 to -1.55)	<0.001
Uzbekistan	6.82（3.32 to 11.44）	0.05（0.02 to 0.09）	22.44（12.17 to 35.58）	0.07（0.04 to 0.11）	1.02 (0.56 to 1.49)	<0.001	283.53（143.13 to 463.57）	1.94（0.97 to 3.20）	886.83（493.59 to 1388.78）	2.61（1.43 to 4.12）	0.92 (0.47 to 1.37)	<0.001
Vanuatu	0.07（0.02 to 0.15）	0.10（0.03 to 0.19）	0.20（0.08 to 0.39）	0.09（0.04 to 0.18）	-0.07 (-0.39 to 0.25)	0.665	2.83（0.93 to 5.62）	3.30（1.08 to 6.55）	7.57（3.10 to 14.77）	3.25（1.33 to 6.31）	-0.03 (-0.38 to 0.31)	0.844
Venezuela (Bolivarian Republic of)	6.68（4.69 to 8.82）	0.06（0.04 to 0.08）	16.53（9.64 to 25.54）	0.05（0.03 to 0.08）	-0.53 (-0.75 to -0.31)	<0.001	240.17（170.46 to 315.24）	1.94（1.37 to 2.55）	562.15（332.05 to 863.17）	1.86（1.10 to 2.86）	-0.21 (-0.43 to 0.01)	0.064
Viet Nam	20.86（1.99 to 48.95）	0.05（0.00 to 0.11）	857.99（452.29 to 1450.79）	0.78（0.41 to 1.32）	9.62 (9.46 to 9.79)	<0.001	847.91（88.56 to 1995.21）	1.75（0.17 to 4.12）	30207.40（15880.44 to 51356.04）	26.65（13.98 to 45.32）	9.23 (9.08 to 9.39)	<0.001
Yemen	1.38（0.54 to 2.90）	0.02（0.01 to 0.05）	0.91（0.35 to 1.89）	0.00（0.00 to 0.01）	-4.92 (-5.18 to -4.66)	<0.001	54.42（20.99 to 115.10）	0.79（0.31 to 1.67）	38.24（14.73 to 78.93）	0.17（0.07 to 0.35）	-4.83 (-5.08 to -4.57)	<0.001
Zambia	10.95（3.83 to 19.21）	0.31（0.11 to 0.54）	53.89（13.91 to 122.59）	0.54（0.15 to 1.20）	1.84 (1.57 to 2.1)	<0.001	425.53（138.93 to 749.72）	10.60（3.70 to 18.58）	2226.80（552.23 to 5124.41）	19.52（5.06 to 44.31）	1.99 (1.73 to 2.25)	<0.001
Zimbabwe	5.11（2.31 to 8.60）	0.11（0.05 to 0.18）	12.39（5.43 to 21.99）	0.13（0.06 to 0.23）	0.75 (0.44 to 1.05)	<0.001	188.44（84.86 to 318.68）	3.52（1.58 to 5.91）	504.02（217.95 to 902.02）	4.84（2.12 to 8.59）	1.09 (0.67 to 1.52)	<0.001

Abbreviations: NPC-AU = Nasopharynx cancer attributable to alcohol use; DALYs = disability-adjusted life years; AAPC = average annual percentage change; ASDR = age-standardized DALYs rate; ASMR = age-standardized mortality rate; SDI = Socio-demographic Index; CI = confidence interval; UI = uncertainty interval.

Table S2. The AAPC of ASDR for NPC-AU at the global level, across the five SDI regions, and within 21 GBD regions, shown for the overall population (A), males (B), and females (C).

Characteristics	AAPC of ASMR (95 % CI)				AAPC of ASDR (95 % CI)			
	Male	P-value	Female	P-value	Male	P-value	Female	P-value
Global	-1.6 (-1.75 to -1.46)	<0.001	-2.25 (-2.39 to -2.1)	<0.001	-1.68 (-1.92 to -1.44)	<0.001	-2.26 (-2.39 to -2.14)	<0.001
High SDI	-1.98 (-2.11 to -1.86)	<0.001	-2.05 (-2.22 to -1.88)	<0.001	-2.23 (-2.34 to -2.12)	<0.001	-2.18 (-2.35 to -2.02)	<0.001
High-middle SDI	-2.02 (-2.17 to -1.86)	<0.001	-2.6 (-2.83 to -2.37)	<0.001	-2.01 (-2.23 to -1.78)	<0.001	-2.6 (-2.84 to -2.35)	<0.001
Low SDI	0.34 (0.13 to 0.56)	0.002	-0.25 (-0.34 to -0.16)	<0.001	0.3 (0.09 to 0.5)	0.005	-0.27 (-0.38 to -0.16)	<0.001
Low-middle SDI	0.88 (0.66 to 1.09)	<0.001	0.41 (0.25 to 0.57)	<0.001	0.79 (0.57 to 1.01)	<0.001	0.41 (0.3 to 0.51)	<0.001
Middle SDI	-1.81 (-1.95 to -1.67)	<0.001	-3.09 (-3.26 to -2.91)	<0.001	-1.92 (-2.03 to -1.8)	<0.001	-3.13 (-3.31 to -2.94)	<0.001
GBD Region								
Andean Latin America	-0.19 (-0.68 to 0.3)	0.441	-0.46 (-1.32 to 0.41)	0.303	-0.32 (-0.83 to 0.19)	0.213	-0.65 (-1.48 to 0.19)	0.13

Australasia	-2.34 (-2.63 to -2.05)	<0.001	-2.08 (-2.41 to -1.76)	<0.001	-2.32 (-2.68 to -1.96)	<0.001	-2.06 (-2.36 to -1.76)	<0.001
Caribbean	0.93 (0.57 to 1.29)	<0.001	0.09 (-0.17 to 0.36)	0.494	0.87 (0.52 to 1.22)	<0.001	0.1 (-0.15 to 0.36)	0.416
Central Asia	0.27 (0.08 to 0.45)	0.005	-0.58 (-0.99 to -0.16)	0.007	0.2 (-0.05 to 0.44)	0.112	-0.7 (-1.17 to -0.22)	0.004
Central Europe	0.1 (-0.07 to 0.26)	0.251	-0.4 (-0.74 to -0.05)	0.026	-0.03 (-0.22 to 0.16)	0.73	-0.5 (-0.87 to -0.12)	0.009
Central Latin America	-0.98 (-1.3 to -0.67)	<0.001	-1.72 (-1.88 to -1.55)	<0.001	-0.98 (-1.28 to -0.68)	<0.001	-1.63 (-1.8 to -1.45)	<0.001
Central Sub-Saharan Africa	0.08 (-0.12 to 0.28)	0.435	-0.29 (-0.57 to -0.02)	0.038	0.06 (-0.13 to 0.25)	0.524	-0.32 (-0.59 to -0.06)	0.018
East Asia	-2.61 (-2.92 to -2.3)	<0.001	-3.9 (-4.17 to -3.63)	<0.001	-2.57 (-2.79 to -2.35)	<0.001	-3.9 (-4.16 to -3.65)	<0.001
Eastern Europe	-0.35 (-1.11 to 0.42)	0.377	0.13 (-0.37 to 0.63)	0.622	-0.4 (-1.15 to 0.36)	0.299	0.14 (-0.36 to 0.64)	0.586
Eastern Sub-Saharan Africa	0.33 (0.26 to 0.41)	<0.001	-0.15 (-0.3 to -0.01)	0.039	0.34 (0.27 to 0.42)	<0.001	-0.24 (-0.38 to -0.09)	0.001
High-income Asia Pacific	-0.67 (-1.07 to -0.28)	0.001	-1.72 (-2.18 to -1.25)	<0.001	-1.15 (-1.43 to -0.87)	<0.001	-1.78 (-2.2 to -1.36)	<0.001
High-income North America	-1.27 (-1.46 to -1.08)	<0.001	-1.57 (-1.89 to -1.25)	<0.001	-1.42 (-1.63 to -1.21)	<0.001	-1.68 (-1.99 to -1.38)	<0.001
North Africa and Middle East	-1.4 (-1.48 to -1.33)	<0.001	-2.78 (-2.95 to -2.61)	<0.001	-1.6 (-1.68 to -1.52)	<0.001	-3.21 (-3.38 to -3.05)	<0.001
Oceania	-0.99 (-1.18 to -0.8)	<0.001	-0.86 (-1.03 to -0.69)	<0.001	-1.03 (-1.19 to -0.87)	<0.001	-0.84 (-1.04 to -0.65)	<0.001
South Asia	0.62 (0.31 to 0.94)	<0.001	-0.07 (-0.29 to 0.15)	0.552	0.5 (0.19 to 0.81)	0.002	-0.08 (-0.3 to 0.13)	0.448
Southeast Asia	1.87 (1.79 to 1.96)	<0.001	0.11 (-0.06 to 0.28)	0.19	1.69 (1.61 to 1.77)	<0.001	0.06 (-0.07 to 0.18)	0.395
Southern Latin America	-3.24 (-3.55 to -2.93)	<0.001	-2.67 (-3.4 to -1.94)	<0.001	-3.27 (-3.62 to -2.92)	<0.001	-2.64 (-3.49 to -1.79)	<0.001
Southern Sub-Saharan Africa	-0.28 (-0.53 to -0.03)	0.031	-0.74 (-1.29 to -0.18)	0.009	-0.29 (-0.54 to -0.04)	0.022	-1.1 (-1.55 to -0.66)	<0.001
Tropical Latin America	0.52 (-0.04 to 1.09)	0.069	1.15 (0.89 to 1.42)	<0.001	0.52 (0.17 to 0.87)	0.004	1.22 (0.89 to 1.55)	<0.001
Western Europe	-2.84 (-2.95 to -2.74)	<0.001	-2.17 (-2.39 to -1.96)	<0.001	-3.02 (-3.13 to -2.91)	<0.001	-2.26 (-2.5 to -2.01)	<0.001
Western Sub-Saharan Africa	-0.04 (-0.13 to 0.05)	0.425	0.15 (-0.01 to 0.3)	0.06	0.04 (-0.06 to 0.14)	0.406	0.12 (-0.05 to 0.3)	0.174

Abbreviations: NPC-AU = Nasopharynx cancer attributable to alcohol use; AAPC = average annual percentage change; ASDR = age-standardized DALYs rate; ASMR = age-standardized mortality rate; SDI = Socio-demographic Index; CI = confidence interval.

Table S3. The actual and predicted values in ASMR and number of deaths cases of NPC-AU.

Year	ASMR (95 % UI)			Number of deaths cases (95 % UI)		
	Both	Male	Female	Both	Male	Female
1990	0.31 （0.00 to 31.85）	0.59 （0.58 to 0.60）	0.05 （0.05 to 0.05）	13466.54 （0.00 to 1625375.79）	12382.95 （0.00 to 802946.19）	1083.59 （0.00 to 822429.60）
1991	0.31 （0.00 to 35.51）	0.60 （0.59 to 0.61）	0.05 （0.05 to 0.05）	13811.14 （0.00 to 1830050.33）	12725.76 （0.00 to 876118.25）	1085.38 （0.00 to 953932.08）
1992	0.31 （0.00 to 36.41）	0.60 （0.59 to 0.61）	0.05 （0.05 to 0.05）	14087.83 （0.00 to 1902171.08）	13001.43 （0.00 to 880230.07）	1086.40 （0.00 to 1021941.01）
1993	0.31 （0.00 to 37.37）	0.59 （0.59 to 0.60）	0.05 （0.05 to 0.05）	14284.45 （0.00 to 1981336.06）	13199.09 （0.00 to 895891.91）	1085.36 （0.00 to 1085444.15）
1994	0.31 （0.00 to 37.79）	0.59 （0.58 to 0.59）	0.05 （0.04 to 0.05）	14373.89 （0.00 to 2035321.96）	13293.30 （0.00 to 906897.40）	1080.58 （0.00 to 1128424.56）
1995	0.30 （0.00 to 38.28）	0.58 （0.57 to 0.58）	0.04 （0.04 to 0.05）	14409.59 （0.00 to 2092723.99）	13338.26 （0.00 to 931020.23）	1071.32 （0.00 to 1161703.75）
1996	0.29 （0.00 to 38.17）	0.56 （0.56 to 0.57）	0.04 （0.04 to 0.04）	14325.97 （0.00 to 2122340.27）	13267.84 （0.00 to 942370.05）	1058.13 （0.00 to 1179970.22）
1997	0.28 （0.00 to 38.07）	0.54 （0.54 to 0.55）	0.04 （0.04 to 0.04）	14168.12 （0.00 to 2154464.45）	13125.98 （0.00 to 956080.41）	1042.15 （0.00 to 1198384.04）
1998	0.27 （0.00 to 38.31）	0.53 （0.52 to 0.53）	0.04 （0.04 to 0.04）	13973.98 （0.00 to 2205496.13）	12949.94 （0.00 to 982177.75）	1024.04 （0.00 to 1223318.38）
1999	0.26 （0.00 to 38.53）	0.51 （0.50 to 0.51）	0.04 （0.04 to 0.04）	13719.80 （0.00 to 2257965.37）	12714.56 （0.00 to 1005878.49）	1005.24 （0.00 to 1252086.88）
2000	0.25 （0.00 to 39.15）	0.49 （0.48 to 0.49）	0.04 （0.04 to 0.04）	13459.24 （0.00 to 2337585.92）	12473.41 （0.00 to 1059959.51）	985.83 （0.00 to 1277626.41）
2001	0.24 （0.00 to 38.79）	0.46 （0.46 to 0.47）	0.04 （0.03 to 0.04）	13153.42 （0.00 to 2358300.97）	12185.95 （0.00 to 1067271.21）	967.46 （0.00 to 1291029.76）

2002	0.23 (0.00 to 38.61)	0.44 (0.44 to 0.45)	0.03 (0.03 to 0.03)	12841.26 (0.00 to 2395970.78)	11890.77 (0.00 to 1098396.84)	950.49 (0.00 to 1297573.93)
2003	0.22 (0.00 to 38.38)	0.42 (0.42 to 0.43)	0.03 (0.03 to 0.03)	12536.52 (0.00 to 2429942.84)	11600.66 (0.00 to 1129438.32)	935.85 (0.00 to 1300504.52)
2004	0.21 (0.00 to 37.84)	0.40 (0.40 to 0.41)	0.03 (0.03 to 0.03)	12309.94 (0.00 to 2439552.69)	11385.21 (0.00 to 1130738.00)	924.73 (0.00 to 1308814.69)
2005	0.20 (0.00 to 37.85)	0.39 (0.39 to 0.40)	0.03 (0.03 to 0.03)	12211.66 (0.00 to 2483849.34)	11294.22 (0.00 to 1142986.31)	917.43 (0.00 to 1340863.03)
2006	0.20 (0.00 to 37.43)	0.38 (0.38 to 0.39)	0.03 (0.03 to 0.03)	12219.61 (0.00 to 2500904.74)	11305.59 (0.00 to 1121712.97)	914.03 (0.00 to 1379191.77)
2007	0.20 (0.00 to 38.14)	0.38 (0.37 to 0.38)	0.03 (0.03 to 0.03)	12409.10 (0.00 to 2590786.32)	11494.61 (0.00 to 1150891.10)	914.49 (0.00 to 1439895.22)
2008	0.20 (0.00 to 38.88)	0.38 (0.37 to 0.38)	0.03 (0.03 to 0.03)	12696.57 (0.00 to 2682363.62)	11778.65 (0.00 to 1178930.98)	917.92 (0.00 to 1503432.64)
2009	0.20 (0.00 to 39.09)	0.38 (0.37 to 0.38)	0.03 (0.03 to 0.03)	13001.95 (0.00 to 2739528.65)	12078.69 (0.00 to 1184529.22)	923.26 (0.00 to 1554999.43)
2010	0.20 (0.00 to 39.14)	0.38 (0.37 to 0.38)	0.03 (0.03 to 0.03)	13318.52 (0.00 to 2786951.41)	12388.92 (0.00 to 1196051.25)	929.60 (0.00 to 1590900.15)
2011	0.20 (0.00 to 38.50)	0.38 (0.37 to 0.38)	0.03 (0.03 to 0.03)	13586.67 (0.00 to 2782636.01)	12650.11 (0.00 to 1178118.46)	936.56 (0.00 to 1604517.55)
2012	0.19 (0.00 to 37.95)	0.38 (0.37 to 0.38)	0.03 (0.03 to 0.03)	13842.58 (0.00 to 2784619.00)	12898.23 (0.00 to 1179571.99)	944.35 (0.00 to 1605047.01)
2013	0.19 (0.00 to 37.84)	0.37 (0.37 to 0.38)	0.03 (0.03 to 0.03)	14067.20 (0.00 to 2818414.96)	13113.62 (0.00 to 1207175.19)	953.59 (0.00 to 1611239.77)
2014	0.19 (0.00 to 38.00)	0.37 (0.37 to 0.37)	0.03 (0.02 to 0.03)	14255.03 (0.00 to 2870022.88)	13290.59 (0.00 to 1246760.58)	964.44 (0.00 to 1623262.30)
2015	0.19 (0.00 to 38.38)	0.37 (0.36 to 0.37)	0.03 (0.02 to 0.03)	14519.03 (0.00 to 2937863.18)	13541.83 (0.00 to 1284883.76)	977.21 (0.00 to 1652979.42)
2016	0.19 (0.00 to 38.80)	0.37 (0.36 to 0.37)	0.03 (0.02 to 0.03)	14815.80 (0.00 to 3009076.73)	13824.87 (0.00 to 1321318.95)	990.93 (0.00 to 1687757.78)
2017	0.19 (0.00 to 38.78)	0.37 (0.36 to 0.37)	0.02 (0.02 to 0.03)	15098.08 (0.00 to 3046256.35)	14092.81 (0.00 to 1329358.62)	1005.27 (0.00 to 1716897.73)
2018	0.19 (0.00 to 38.69)	0.36 (0.36 to 0.37)	0.02 (0.02 to 0.03)	15384.92 (0.00 to 3076141.63)	14364.90 (0.00 to 1330158.77)	1020.01 (0.00 to 1745982.86)
2019	0.19 (0.00 to 38.57)	0.36 (0.36 to 0.37)	0.02 (0.02 to 0.03)	15706.50 (0.00 to 3102404.72)	14671.51 (0.00 to 1334582.23)	1035.00 (0.00 to 1767822.49)
2020	0.19 (0.00 to 38.28)	0.36 (0.36 to 0.37)	0.02 (0.02 to 0.03)	16034.89 (0.00 to 3110042.90)	14985.86 (0.00 to 1338233.75)	1049.03 (0.00 to 1771809.15)
2021	0.19 (0.00 to 38.29)	0.36 (0.36 to 0.37)	0.02 (0.02 to 0.03)	16329.57 (0.00 to 3133760.77)	15267.72 (0.00 to 1333488.91)	1061.85 (0.00 to 1800271.86)
2022	0.19 (0.00 to 48.34)	0.36 (0.35 to 0.38)	0.02 (0.02 to 0.03)	16810.02 (0.00 to 4092994.92)	15732.86 (0.00 to 1801945.37)	1077.16 (0.00 to 2291049.56)
2023	0.19 (0.00 to 81.65)	0.36 (0.34 to 0.38)	0.02 (0.02 to 0.03)	17027.13 (0.00 to 7111781.52)	15990.84 (0.00 to 3322266.39)	1036.29 (0.00 to 3789515.13)
2024	0.18 (0.00 to 142.38)	0.36 (0.33 to 0.39)	0.02 (0.02 to 0.03)	16957.94 (0.00 to 12685866.86)	16136.75 (0.00 to 5751150.91)	821.19 (0.00 to 6934715.95)
2025	0.17 (0.00 to 210.91)	0.36 (0.32 to 0.40)	0.02 (0.02 to 0.03)	16586.64 (0.00 to 19101292.49)	16228.46 (0.00 to 7956886.93)	358.18 (0.00 to 11144405.56)
2026	0.17 (0.00 to 274.95)	0.36 (0.31 to 0.42)	0.02 (0.02 to 0.03)	16416.37 (0.00 to 25241248.22)	16336.04 (0.00 to 9632318.83)	80.34 (0.00 to 15608929.38)
2027	0.17 (0.00 to 330.06)	0.36 (0.29 to 0.43)	0.02 (0.02 to 0.03)	16515.53 (0.00 to 30677351.19)	16484.34 (0.00 to 10847977.48)	31.19 (0.00 to 19829373.71)
2028	0.16 (0.00 to 375.94)	0.36 (0.28 to 0.44)	0.02 (0.02 to 0.03)	16688.39 (0.00 to 35355689.86)	16671.58 (0.00 to 11747011.87)	16.81 (0.00 to 23608677.98)
2029	0.16 (0.00 to 413.39)	0.36 (0.26 to 0.46)	0.02 (0.02 to 0.03)	16907.12 (0.00 to 39328156.69)	16897.26 (0.00 to 12420819.94)	9.86 (0.00 to 26907336.74)
2030	0.16 (0.00 to 443.52)	0.36 (0.24 to 0.47)	0.02 (0.02 to 0.03)	17168.68 (0.00 to 42674448.68)	17162.70 (0.00 to 12923248.36)	5.98 (0.00 to 29751200.32)
2031	0.16 (0.00 to 467.43)	0.36 (0.23 to 0.49)	0.02 (0.01 to 0.03)	17470.53 (0.00 to 45478643.46)	17466.32 (0.00 to 13289877.47)	4.20 (0.00 to 32188765.98)

2032	0.16<br>（0.00 to 486.19）	0.36<br>（0.21 to 0.51）	0.02<br>（0.01 to 0.03）	17807.70<br>（0.00 to 47824320.25）	17805.50<br>（0.00 to 13549558.02）	2.20<br>（0.00 to 34274762.23）
2033	0.16<br>（0.00 to 500.77）	0.36<br>（0.19 to 0.53）	0.02<br>（0.01 to 0.03）	18178.27<br>（0.00 to 49788331.94）	18178.15<br>（0.00 to 13729715.32）	0.11<br>（0.00 to 36058616.62）
2034	0.16<br>（0.00 to 511.88）	0.36<br>（0.17 to 0.55）	0.02<br>（0.01 to 0.03）	18586.40<br>（0.00 to 51433071.71）	18586.40<br>（0.00 to 13848232.35）	0.00<br>（0.00 to 37584839.35）
2035	0.17<br>（0.00 to 520.04）	0.36<br>（0.15 to 0.57）	0.02<br>（0.01 to 0.03）	19032.75<br>（0.00 to 52798468.82）	19032.75<br>（0.00 to 13912690.65）	0.00<br>（0.00 to 38885778.17）
2036	0.17<br>（0.00 to 525.67）	0.36<br>（0.13 to 0.59）	0.02<br>（0.01 to 0.03）	19518.07<br>（0.00 to 53914638.68）	19518.07<br>（0.00 to 13927568.10）	0.00<br>（0.00 to 39987070.58）
2037	0.17<br>（0.00 to 529.12）	0.36<br>（0.10 to 0.61）	0.02<br>（0.01 to 0.04）	20042.88<br>（0.00 to 54809477.71）	20042.88<br>（0.00 to 13897771.10）	0.00<br>（0.00 to 40911706.60）
2038	0.17<br>（0.00 to 530.73）	0.35<br>（0.08 to 0.63）	0.02<br>（0.01 to 0.04）	20609.96<br>（0.00 to 55509739.97）	20609.96<br>（0.00 to 13830763.64）	0.00<br>（0.00 to 41678976.33）
2039	0.17<br>（0.00 to 530.74）	0.35<br>（0.06 to 0.65）	0.02<br>（0.01 to 0.04）	21224.52<br>（0.00 to 56038101.23）	21224.52<br>（0.00 to 13731914.38）	0.00<br>（0.00 to 42306186.85）
2040	0.18<br>（0.00 to 529.34）	0.35<br>（0.03 to 0.67）	0.02<br>（0.00 to 0.04）	21889.86<br>（0.00 to 56407554.53）	21889.86<br>（0.00 to 13602925.92）	0.00<br>（0.00 to 42804628.61）

Abbreviations: NPC-AU = Nasopharynx cancer attributable to alcohol use; ASMR = age-standardized mortality rate; UI = uncertainty interval.

Table S4. The actual and predicted values in ASDR and number of deaths cases of NPC-AU.

Year	ASDR			Number of DALYs cases (95 % UI)		
	Both	Male	Female	Both	Male	Female
1990	11.00<br>（5.46 to 23.91）	20.55<br>（20.49 to 20.61）	1.68<br>（1.58 to 1.77）	493806.75<br>（235026.71 to 1093639.43）	455695.64<br>（211110.73 to 901007.81）	38111.11<br>（23915.98 to 192631.62）
1991	11.12<br>（5.58 to 23.41）	20.78<br>（20.72 to 20.84）	1.67<br>（1.58 to 1.76）	509957.21<br>（245699.03 to 1098932.18）	471250.67<br>（220741.07 to 925180.59）	38706.53<br>（24957.95 to 173751.59）
1992	11.08<br>（5.57 to 23.10）	20.72<br>（20.66 to 20.78）	1.65<br>（1.56 to 1.73）	518839.09<br>（250605.19 to 1111311.44）	479851.63<br>（225212.85 to 942458.44）	38987.46<br>（25392.34 to 168853.00）
1993	11.00<br>（5.50 to 22.85）	20.57<br>（20.52 to 20.63）	1.63<br>（1.54 to 1.71）	525653.40<br>（252199.12 to 1126035.69）	486295.83<br>（226459.66 to 957745.65）	39357.57<br>（25739.46 to 168290.04）
1994	10.81<br>（5.32 to 22.52）	20.23<br>（20.17 to 20.28）	1.60<br>（1.52 to 1.68）	527195.06<br>（248754.26 to 1136495.68）	487799.62<br>（222993.39 to 967693.27）	39395.44<br>（25760.87 to 168802.41）
1995	10.64<br>（5.19 to 22.33）	19.92<br>（19.87 to 19.98）	1.56<br>（1.48 to 1.64）	528908.23<br>（247681.58 to 1153096.34）	489749.30<br>（222182.92 to 979240.26）	39158.93<br>（25498.66 to 173856.08）
1996	10.33<br>（4.93 to 22.07）	19.35<br>（19.30 to 19.41）	1.50<br>（1.42 to 1.58）	523775.01<br>（239857.24 to 1166352.75）	485289.48<br>（214962.79 to 984674.20）	38485.52<br>（24894.45 to 181678.55）
1997	9.96<br>（4.61 to 21.83）	18.69<br>（18.64 to	1.43<br>（1.36 to 1.51）	516193.87<br>（229126.35 to	478570.51<br>（204983.02 to	37623.36<br>（24143.33 to

		18.75）		1179775.21）	987442.48）	192332.73）
1998	9.62（4.33 to 21.66）	18.06（18.01 to 18.11）	1.38（1.31 to 1.45）	508806.90（219462.89 to 1196242.70）	471951.27（196010.67 to 990039.34）	36855.63（23452.22 to 206203.36）
1999	9.22（3.99 to 21.49）	17.31（17.26 to 17.36）	1.32（1.26 to 1.39）	498391.94（206604.18 to 1213823.09）	462166.94（183730.55 to 990873.45）	36225.00（22873.62 to 222949.63）
2000	8.89（3.67 to 21.36）	16.68（16.64 to 16.73）	1.27（1.21 to 1.34）	490666.15（194236.96 to 1234116.07）	455081.27（171953.25 to 996194.36）	35584.88（22283.71 to 237921.70）
2001	8.46（3.31 to 21.12）	15.89（15.84 to 15.94）	1.22（1.16 to 1.28）	477541.37（179020.56 to 1247995.10）	442686.31（157442.33 to 996429.81）	34855.06（21578.23 to 251565.29）
2002	8.07（2.96 to 20.89）	15.14（15.10 to 15.19）	1.17（1.11 to 1.23）	465967.25（164158.96 to 1262954.98）	431890.34（143403.09 to 997565.71）	34076.91（20755.88 to 265389.28）
2003	7.68（2.62 to 20.67）	14.41（14.36 to 14.45）	1.11（1.06 to 1.17）	453985.80（148563.79 to 1277692.74）	420657.46（128649.18 to 999760.16）	33328.35（19914.61 to 277932.58）
2004	7.33（2.35 to 20.46）	13.76（13.72 to 13.80）	1.07（1.02 to 1.13）	443511.61（136587.99 to 1291767.00）	410726.99（117430.00 to 1003241.08）	32784.61（19157.99 to 288525.93）
2005	7.10（2.19 to 20.29）	13.33（13.29 to 13.37）	1.04（0.98 to 1.09）	439691.40（130576.71 to 1307273.15）	407166.61（111870.75 to 1013574.84）	32524.79（18705.96 to 293698.30）
2006	6.89（2.04 to 20.01）	12.94（12.90 to 12.98）	1.01（0.96 to 1.06）	436817.44（125156.58 to 1314387.75）	404447.79（106660.07 to 1023431.60）	32369.65（18496.51 to 290956.15）
2007	6.85（2.02 to 20.00）	12.88（12.84 to 12.92）	0.99（0.94 to 1.04）	444440.82（127449.40 to 1336736.26）	411823.88（108958.26 to 1042612.04）	32616.94（18491.13 to 294124.22）
2008	6.85（2.03 to 20.06）	12.90（12.86 to 12.94）	0.98（0.93 to 1.03）	454910.47（131276.06 to 1363285.58）	421934.42（112663.04 to 1062929.95）	32976.05（18613.01 to 300355.63）
2009	6.83（2.01 to 20.10）	12.87（12.84 to 12.91）	0.97（0.92 to 1.02）	463647.36（133452.08 to 1389424.69）	430337.39（114831.14 to 1080760.47）	33309.97（18620.93 to 308664.22）
2010	6.82（2.01 to 20.11）	12.87（12.83 to 12.90）	0.95（0.90 to 1.00）	473347.94（137325.57 to 1412685.50）	439793.38（118723.78 to 1099040.33）	33554.57（18601.79 to 313645.17）
2011	6.74（1.99 to 20.08）	12.75（12.71 to 12.78）	0.93（0.88 to 0.98）	478789.12（140246.70 to 1432624.98）	445286.91（121633.27 to 1114111.13）	33502.21（18613.43 to 318513.84）
2012	6.68（1.99 to 19.99）	12.65（12.61 to 12.69）	0.90（0.86 to 0.95）	484934.41（143662.53 to 1448204.74）	451577.00（125027.77 to 1129692.39）	33357.41（18634.75 to 318512.35）
2013	6.63（1.93 to 19.98）	12.56（12.52 to 12.60）	0.89（0.84 to 0.93）	491688.89（143591.28 to 1469688.90）	458308.03（124829.19 to 1145966.58）	33380.87（18762.09 to 323722.33）
2014	6.56（1.82 to 20.01）	12.44（12.41 to 12.48）	0.87（0.83 to 0.92）	497374.52（138678.37 to 1493194.13）	463730.83（119595.14 to 1161461.46）	33643.69（19083.24 to 331732.67）
2015	6.54（1.79 to 20.07）	12.41（12.37 to 12.44）	0.87（0.82 to 0.91）	506077.43（139767.62 to 1518226.68）	471965.16（120283.32 to 1178475.67）	34112.28（19484.29 to 339751.02）
2016	6.54（1.78 to 20.35）	12.41（12.38 to 12.45）	0.87（0.82 to 0.91）	516510.31（142617.82 to 1559899.16）	481810.83（122698.00 to 1196441.57）	34699.48（19919.82 to 363457.59）
2017	6.52（1.76 to 20.65）	12.37（12.33 to 12.40）	0.86（0.82 to 0.90）	524864.90（144384.77 to 1603243.45）	489739.78（124199.86 to 1212161.96）	35125.12（20184.91 to 391081.48）

2018	6.49 (1.73 to 21.38)	12.31 (12.28 to 12.35)	0.85 (0.81 to 0.90)	532575.69 (145251.34 to 1678375.56)	497099.71 (124848.69 to 1227537.40)	35475.98 (20402.65 to 450838.16)
2019	6.47 (1.73 to 23.07)	12.29 (12.26 to 12.32)	0.84 (0.80 to 0.89)	541660.84 (148996.03 to 1826477.09)	505854.66 (128166.62 to 1244128.14)	35806.18 (20829.41 to 582348.94)
2020	6.45 (1.75 to 26.47)	12.26 (12.23 to 12.29)	0.84 (0.79 to 0.88)	550129.43 (153433.03 to 2103515.02)	514047.85 (132272.08 to 1259024.90)	36081.58 (21160.95 to 844490.12)
2021	6.45 (1.73 to 33.34)	12.26 (12.23 to 12.30)	0.83 (0.79 to 0.88)	558992.54 (155141.32 to 2648394.51)	522459.92 (134312.96 to 1275587.17)	36532.62 (20828.37 to 1372807.34)
2022	6.39 (0.68 to 84.61)	12.16 (11.76 to 12.57)	0.83 (0.76 to 0.90)	570629.95 (63868.61 to 6957208.08)	533706.11 (63868.61 to 1555354.74)	36923.84 (0.00 to 5401853.33)
2023	6.37 (0.00 to 113.59)	12.12 (11.47 to 12.78)	0.82 (0.73 to 0.91)	578645.48 (0.00 to 9557141.62)	541429.92 (0.00 to 2239465.69)	37215.56 (0.00 to 7317675.93)
2024	6.35 (0.00 to 117.03)	12.08 (11.10 to 13.06)	0.82 (0.72 to 0.91)	586715.25 (0.00 to 10128740.39)	548902.80 (0.00 to 3646217.33)	37812.45 (0.00 to 6482523.07)
2025	6.33 (0.00 to 104.11)	12.04 (10.69 to 13.39)	0.81 (0.70 to 0.92)	594688.35 (0.00 to 9178791.61)	556283.46 (0.00 to 5078229.02)	38404.89 (0.00 to 4100562.59)
2026	6.31 (0.00 to 97.79)	11.99 (10.23 to 13.76)	0.81 (0.68 to 0.93)	602620.00 (0.00 to 8682436.10)	563737.22 (0.00 to 6165464.97)	38882.78 (0.00 to 2516971.12)
2027	6.29 (0.00 to 132.38)	11.95 (9.74 to 14.16)	0.80 (0.66 to 0.94)	610590.89 (0.00 to 11888647.55)	571445.79 (0.00 to 6904407.93)	39145.09 (0.00 to 4984239.62)
2028	6.28 (0.00 to 187.24)	11.91 (9.22 to 14.61)	0.80 (0.64 to 0.96)	618515.81 (0.00 to 17115297.91)	579487.06 (0.00 to 7400974.72)	39028.75 (0.00 to 9714323.19)
2029	6.27 (0.00 to 220.42)	11.87 (8.67 to 15.08)	0.79 (0.61 to 0.98)	626608.03 (0.00 to 20459979.56)	587692.19 (0.00 to 7736326.38)	38915.84 (0.00 to 12723653.18)
2030	6.27 (0.00 to 237.00)	11.83 (8.09 to 15.57)	0.79 (0.57 to 1.00)	635088.04 (0.00 to 22319080.00)	596103.14 (0.00 to 7958904.04)	38984.90 (0.00 to 14360175.96)
2031	6.27 (0.00 to 242.12)	11.79 (7.49 to 16.09)	0.79 (0.54 to 1.03)	644182.11 (0.00 to 23118607.08)	604912.07 (0.00 to 8095671.57)	39270.04 (0.00 to 15022935.51)
2032	6.28 (0.00 to 240.26)	11.75 (6.87 to 16.64)	0.78 (0.50 to 1.06)	654124.02 (0.00 to 23249589.78)	614396.73 (0.00 to 8165123.97)	39727.30 (0.00 to 15084465.81)
2033	6.31 (0.00 to 234.68)	11.73 (6.23 to 17.22)	0.78 (0.46 to 1.10)	665029.58 (0.00 to 23004848.52)	624727.48 (0.00 to 8185270.51)	40302.10 (0.00 to 14819578.00)
2034	6.34 (0.00 to 227.32)	11.70 (5.57 to 17.82)	0.78 (0.42 to 1.13)	676680.63 (0.00 to 22568859.11)	635722.38 (0.00 to 8170039.66)	40958.25 (0.00 to 14398819.46)
2035	6.38 (0.00 to 219.27)	11.67 (4.89 to 18.44)	0.77 (0.38 to 1.17)	689001.73 (0.00 to 22042505.47)	647329.90 (0.00 to 8125819.74)	41671.83 (0.00 to 13916685.73)
2036	6.43 (0.00 to 211.04)	11.64 (4.19 to 19.08)	0.77 (0.34 to 1.21)	702177.89 (0.00 to 21476588.35)	659739.38 (0.00 to 8055339.38)	42438.51 (0.00 to 13421248.97)
2037	6.49 (0.00 to 202.90)	11.61 (3.47 to 19.75)	0.77 (0.29 to 1.25)	716513.92 (0.00 to 20897020.45)	673256.14 (0.00 to 7960967.50)	43257.78 (0.00 to 12936052.95)
2038	6.56 (0.00 to	11.59 (2.74 to 20.45)	0.77 (0.25 to 1.29)	732218.11 (0.00 to	688087.92 (0.00 to	44130.20 (0.00 to

	195.00 ）			20321493.29 ）	7847211.60 ）	12474281.68 ）
2039	6.64 （0.00 to 187.42 ）	11.57 （1.98 to 21.16 ）	0.77 （0.20 to 1.33 ）	749162.00 （0.00 to 19759895.60 ）	704098.85 （0.00 to 7718516.82 ）	45063.15 （0.00 to 12041378.78 ）
2040	6.74 （0.00 to 180.16 ）	11.55 （1.21 to 21.89 ）	0.77 （0.15 to 1.38 ）	767302.30 （0.00 to 19212871.95 ）	721242.06 （0.00 to 7576973.90 ）	46060.25 （0.00 to 11635898.05 ）

Abbreviations: NPC-AU = Nasopharynx cancer attributable to alcohol use; DALYs = disability-adjusted life years; ASDR = age-standardized DALYs rate; ASMR = age-standardized mortality rate;c UI = uncertainty interval.
